# A comparative study in class imbalance mitigation when working with physiological signals

**DOI:** 10.3389/fdgth.2024.1377165

**Published:** 2024-03-26

**Authors:** Rawan S. Abdulsadig, Esther Rodriguez-Villegas

**Affiliations:** Wearable Technologies Lab, Department of Electrical and Electronic Engineering, Imperial College London, London, United Kingdom

**Keywords:** class imbalance, machine learning, physiological signals, sudden unexpected death in epilepsy (SUDEP), apnea

## Abstract

Class imbalance is a common challenge that is often faced when dealing with classification tasks aiming to detect medical events that are particularly infrequent. Apnoea is an example of such events. This challenge can however be mitigated using class rebalancing algorithms. This work investigated 10 widely used data-level class imbalance mitigation methods aiming towards building a random forest (RF) model that attempts to detect apnoea events from photoplethysmography (PPG) signals acquired from the neck. Those methods are random undersampling (RandUS), random oversampling (RandOS), condensed nearest-neighbors (CNNUS), edited nearest-neighbors (ENNUS), Tomek’s links (TomekUS), synthetic minority oversampling technique (SMOTE), Borderline-SMOTE (BLSMOTE), adaptive synthetic oversampling (ADASYN), SMOTE with TomekUS (SMOTETomek) and SMOTE with ENNUS (SMOTEENN). Feature-space transformation using PCA and KernelPCA was also examined as a potential way of providing better representations of the data for the class rebalancing methods to operate. This work showed that RandUS is the best option for improving the sensitivity score (up to 11%). However, it could hinder the overall accuracy due to the reduced amount of training data. On the other hand, augmenting the data with new artificial data points was shown to be a non-trivial task that needs further development, especially in the presence of subject dependencies, as was the case in this work.

## 1 Introduction

Class imbalance is one of the most challenging problems when training machine learning models, especially when data acquisition is expensive. The problem of class imbalance arises when some classes (or categories) have significantly smaller number of samples compared to others, leading to a model that is less likely to detect those minority classes due to the insufficient number of samples in the training set needed for proper learning. This problem presents itself in various domains and applications including but not limited to security, finance, environment, agriculture, and health (1–4). Typically, class imbalance is mitigated either at the model level by adapting and adjusting the training procedure based on the different data samples and training progression, or at the data level by modifying the class distributions in such a way as to allow for improved class separability, typically via resampling (5–7). Resampling techniques are widely used in the literature. Those include undersampling techniques that attempt to change the distribution of the majority classes such as random undersampling (RandUS), condensed nearest-neighbors (CNN), edited nearest-neighbors (ENN), and Tomek’s links (Tomek), as well as oversampling techniques that change the distribution of the minority classes such as random oversampling (RandOS), synthetic minority oversampling (SMOTE), Borderline-SMOTE (BLSMOTE), and adaptive synthetic oversampling (ADASYN). In addition to hybrid resampling techniques where a combination of undersampling and oversampling methods are applied in unison.

A study investigating the effectiveness of rebalancing imbalanced datasets prior to developing predictive models, analyzed the effect of four resampling techniques on 17 different datasets obtained from the UCI Machine Learning Database Repository, employing eight classical machine learning classifiers. The study concluded that, in general, oversampling was found to perform better than undersampling, due to the reduced number of data points when performing undersampling which could take away useful information from the training process (8). On the other hand, a later extensive review of the available methods for learning from imbalanced datasets showed that no specific method was found to consistently outperform the rest, and that their performance differs greatly depending on the type of data and application (3).

When it comes to building machine-learning models for medical applications, class imbalance is a typical challenge where the positive class of concern (the event or the condition) represents a rare or infrequent occurrence in the data, while the negative class (the absence of the event or condition) represents the majority of occurrences in the data. Diabetes diagnosis is an example application where class imbalance can occur, and a recent study attempted to tackle this problem in order to improve the performance of machine learning models (9). In that work, the dataset consisted of many nominal patient attributes such as BMI, age, and marital status. ENN, SMOTE, SMOTEENN and SMOTETomek were investigated, and it was found that undersampling using ENN resulted in superior improvements, especially in terms of recall, while the hybrid methods produced less but comparable improvements. Another recent study attempted to mitigate the effect of class imbalance on apnoea detection using SMOTE, SVM-SMOTE, Kmeans-SMOTE, SMOTEENN, SMOTETomek among other methods, including ensemble-based methods, concluded that using a combination of random undersampling and duplicative oversampling gave superior improvements (10). The data in that work was obtained from the St. Vincent’s University Hospital/University College Dublin Sleep Apnea Database, and the features used were the SpO2 level, SpO2 drop, duration of the event in addition to the BMI and the Epworth sleepiness score. The results of that study suggest that adjusting the distributions of the classes using simple methods can be superior to the other more sophisticated methods available, in certain applications.

Detecting Apnoea occurrences in real-time is particularly important for the prevention of Sudden Unexpected Death in Epilepsy (SUDEP). A retrospective study (MORTEMUS) that comprehensively evaluated data obtained from various epilepsy monitoring units revealed the circumstances that proceeded to the tragic SUDEP events. The study showed the presence of transient apnoea episodes which developed within 3 min after a generalized tonic-clonic seizure, pointing to cardiorespiratory dysfunction that eventually led to terminal apnoea and cardiac arrest, tragically ending the life of the patient (11). This could suggest that the ability to immediately detect and alert when an apnoea event occurs can help prevent further complications and potentially save lives, highlighting the importance of developing accurate and reliable apnoea detection methods. The speedy real-time apnoea alert requires quick responses to physiological cues, and bypassing the need for obtaining calibrated measurements such as SpO2 [as seen in previous studies (10, 12, 13)] could allow for increased speed of response, since SpO2 is measured by calibrating photoplethysmography (PPG) readings. The raw PPG waveform is rarely used to detect apnoea, however, it was proven to be feasible especially when obtained from the neck since it becomes strongly modulated by respiration (14). Indeed it was shown in a later study that it was possible to detect apnoea events using PPG signals without the need for obtaining SpO2 measurements that pose a significant time delay (15).

The focus of this work is to carry out an extensive comparative evaluation of the most widely used data-level undersampling, oversampling, and hybrid algorithms for class rebalancing. This was done while using the detection of apnoeas from PPG signals as the target application.

## 2 Materials and methods

In this work, ten well-known and widely used class rebalancing methods were examined in the classification task of detecting apnoea vs non-apnoea PPG segments, where PPG signals were acquired from the neck. Four of these are undersampling methods, four more are oversampling methods, and two hybrid methods which perform both undersampling and oversampling. PPG signals were preprocessed, features were extracted and classes were annotated. An optional step was examined where dimensionality reduction using PCA was applied to the feature space, in an attempt to provide the class rebalancing methods with a different and possibly better spatial domain to operate. Random forest (RF) was the classification model of choice due to its extensive use in literature and frequent superiority over other classical machine-learning algorithms. The following subsections provide further description of the methodology behind this work.

### 2.1 Data acquisition

PPG data was obtained using an in-house customized device integrating a reflectance PPG sensor (MAX30102, MAXIM integrated) that emits red and IR light (650–670 nm and 870–900 nm, respectively) with an NRF5232 microcontroller (Nordic Semiconductor) along with a rechargeable 3.8 V 80 mAh lithium polymer battery. The PPG data was sampled at 400 Hz and transmitted wirelessly via Bluetooth low energy (BLE) to a locally developed data acquisition iOS app. The device was placed approximately 1 in above the suprasternal notch on the neck using a double-sided adhesive tape which is shaped in a way not to obstruct the PPG light trajectory. This setup was followed in previous work (16).

In this work, 8 healthy participants were recruited (5 males and 3 females) as part of a study approved by the Local Ethics Committee of Imperial College London (ICREC reference number: 18IC4358). Table 1 lists the main details of those subjects.

**Table 1:** Demographic details of the participating subjects.

	Mean value ± std
Age	27±2.8 years
Height	175.88±8.2 cm
Weight	69.2±12.10 kg
BMI	22.45±4.26 (${\mathrm{kg}/m}^{2}$)

During the experiment, all participants were directed to hold their breath at different times following verbal cues, and for as long as they could without overly forcing their bodies, as that could lead to involuntary reflexes resulting in non-realistic artifacts. Subjects signaled the beginning and end of each breath held by gently raising their hand. This was done to ensure precise marking of the duration of time the apnoea event was simulated. During each data acquisition session, artifacts provoked by the subjects such as talking or excessive movement as well as any mislabelling of apnoea events were marked immediately for later elimination. Each subject was asked to hold their breath between 3 to 10 times, within a ≈ 30-min. data acquisition run. The duration of each apnoea event in the data was between 10 to 100 s.

### 2.2 Data preprocessing

The PPG’s Red and IR channels were downsampled to 100 Hz, and segmented using a 30 s long overlapping sliding window shifting by 1 s.

Signals captured within each window were first filtered using a median filter with a window of 5 samples in order to remove transient noise in the PPG channels, then a smoothing 2nd order Savitsky-Golay filter with a window of size 0.25 s. The independently filtered Red and IR channels were finally combined by time-wise addition and then standardized, resulting in a unified signal ready to be used for feature extraction.

### 2.3 Feature extraction and dataset construction

A total of 49 features were extracted from each preprocessed PPG segment, all of which were proposed and evaluated for processing PPG signals in previous studies (15, 17), Tables 2, 3 list those features and their brief description.

**Table 2:** Time-domain features extracted from PPG pulses.

Feature	Description
Pulse amplitude	The vertical distance between the onset and the systolic peak of a PPG pulse.
Pulse width	The duration of time (in seconds) between the onset and the offset of a PPG pulse.
Pulse height difference	Difference in amplitude between successive PPG pulse peaks.
Pulse distance	Difference in time (in seconds) between successive PPG pulse peaks.
Trough difference	Difference in onset amplitude of successive PPG pulses.
Rise time	Duration of time (in seconds) between the onset and the systolic peak of a PPG pulse.
Skewness	Level of asymmetry of a PPG pulse.
Kurtosis	Level of non-Gaussian behavior of a PPG pulse.

**Table 3:** Frequency-domain, correlogram and envelope features extracted from PPG segments.

Feature	Description
Spectral entropy	The level of irregularity of power in the frequency domain. This value was calculated for the frequency ranges [0,1.5] Hz and [1,4] Hz.
Spectral kurtosis	The level of peakedness or non-Gaussian behavior in the frequency domain. This value was calculated for the frequency bands [0,1.5] Hz and [1,4] Hz
Relative power	A ratio of the power within a specified range to the total power across all frequencies. This measure was calculated for the frequency bands [0,0.8] Hz, [0.8,1.3] Hz and [1.3,1.8] Hz
Average band power	The mean power within a specific range of frequencies. This measure was calculated for the frequency bands [0,0.8] Hz, [0.8,1.3] Hz, [1.3,1.8] Hz, [2.2,2.8] Hz and [3.2,3.8] Hz
Correlogram peaks	The value of peak of the autocorrelation function. This was calculated for the first and second peaks.
Correlogram lags	The amount of lag a peak in the autocorrelation function. This was calculated for the first and second peaks.
Envelope statistics	Standard deviation, maximum and minimum values of the envelope.
Envelope range	The difference of the maximum and minimum values of the envelope.
Envelope area	Area under the envelope’s absolute values calculated numerically using the trapezoidal method.
Envelope average power	The mean power of the envelope within certain frequency ranges. This value was calculated for the frequency bands [0,0.15] Hz, [0.2,0.5] Hz, [0,0.5] Hz and [0.5,1] Hz
Envelope approximate entropy	The level of irregularity in the envelope. This was calculated using Python’s “EntropyHub.ApEn” function.

Table 2 lists the time-domain features that were extracted per PPG pulse, from which the mean, standard deviation and mean difference between consecutive pulses were calculated per window segment. The PPG pulses were obtained using the approach described in (18) and provided in their GiHub repository (https://github.com/akrlowicz/ppg-blood-pressure-estimation). Table 3 shows the frequency-domain, correlogram and envelop features that were calculated over the window segments. The envelope features were obtained from the upper envelope of the PPG signal as described in (15). Features were standardized before further processing, and standardization parameters were strictly obtained from the training partition of the dataset. Each window segment was labeled as apnoea if it carried at least 5 s worth of apnoea annotated samples, otherwise, it was labeled as non-apnoea.

### 2.4 Class rebalancing methods

Class imbalance is typically treated either by undersampling the majority-class data points or oversampling the minority-class data points in such a way as to lessen or eliminate the difference in the number of data points belonging to each. Furthermore, hybrid approaches applying both undersampling and oversampling are widely used to achieve the same objective, with potentially cleaner class distributions. In this work, the implementations provided by the Python library “imblearn” were used to execute 4 undersampling methods, 4 oversampling methods and 2 hybrid methods. A brief description of each method is given below.

#### 2.4.1 Random undersampling (RandUS)

In random undersampling (RandUS), the majority class is undersampled by randomly selecting a subset of data points by a factor corresponding to the number of minority class data points.

#### 2.4.2 Random oversampling (RandOS)

In random oversampling (RandOS), the minority class is augmented by randomly selecting and duplicating data points from that class, which could be thought of as increasing the weight of the minority class samples by a factor proportional to the number of duplicates per data point.

#### 2.4.3 Condensed nearest-neighbors undersampling (CNNUS)

The objective of the condensed nearest-neighbors undersampling (CNNUS) method is to only consider the most influential points from the majority class. It was originally proposed as a way to reduce the number of data points needed to be stored in memory for nearest-neighbor classifiers (19). However, it was then widely used as a class rebalancing undersampling approach.

The way to determine an influential point that is worth keeping is by iteratively going over sampled points from the majority class and using the K nearest-neighbors (KNN) classification algorithm in order to classify that point with respect to the minority class points and the remaining majority class points. In case it was misclassified, then it means that it is an influential point that needs to be retained. Should it be classified correctly then it would be considered as a redundant point and would be removed. K is typically defined as 1, although it is a tunable parameter.

#### 2.4.4 Edited nearest-neighbors undersampling (ENNUS)

In the edited nearest-neighbors undersampling (ENNUS) method, for a majority data point to be kept in the dataset, its K nearest-neighbors have to also belong to the majority class (20). Two methods of evaluation are typically used; majority voting or complete agreement. The former only requires most of the K neighbors to share its class while the latter requires all of them to do so, which makes the latter more strict than the former while counteracting the possibility of having rare and scattered minority class points, therefore, the latter was used to represent the method.

#### 2.4.5 Tomek’s links undersampling (TomekUS)

The Tomek’s links undersampling (TomekUS) method attempts to remove data points from the majority class that exhibit a Tomek’s link (21). A Tomek’s link occurs when a minority-class data point x and a majority-class data point y are both the nearest-neighbors of each other, such that for any other data point z:

dist(x,y)<dist(x,z) and dist(x,y)<dist(y,z)

Where dist(a,b) is the distance between point $a\in {ℜ}^{n}$ and $b\in {ℜ}^{n}$, which is often represented by the Euclidean distance.

#### 2.4.6 Synthetic minority oversampling technique (SMOTE)

The synthetic minority oversampling technique (SMOTE) algorithm aims to augment the minority class points by generating new data points that are composed of a random linear combination of pairs of minority data points within a certain neighborhood of K points (22). In this method, the number of neighbors within the K nearest-neighbors region to be used as anchors for generation depends on the amount of oversampling required, therefore, K does not influence the number of generated data points but the size of the regions from which the pairs are made.

#### 2.4.7 Borderline-SMOTE (BLSMOTE)

Borderline-SMOTE (BLSMOTE) is a variation of the SMOTE algorithm which focuses on the minority-class data points closest to the majority-class region, i.e., borderline, and only those data points are oversampled (5). Two variations were proposed: Borderline-SMOTE1 and Borderline-SMOTE2. Borderline-SMOTE1 oversamples minority points that reside in the “DANGER” set which is defined by having more majority-class neighbors than minority-class’s, while the number of majority-class data points is not equal to the number of neighbors K. Borderline-SMOTE2 differs from Borderline-SMOTE1 in that it does not only generate new samples based on minority-class nearest-neighbors pairs, but it also pairs minority-class points with their nearest majority-class neighbors and forms a random linear combination that leans towards the minority-class side of the pair. Borderline-SMOTE1 was chosen to represent this method in this work.

#### 2.4.8 Adaptive synthetic oversampling (ADASYN)

The adaptive synthetic oversampling (ADASYN) method, like SMOTE, aims to generate new minority data points along the lines connecting pairs of minority data points within a K nearest-neighbors region. Unlike SMOTE, it uses the density distribution of the majority-class data points around each minority-class data point to determine the proportion of synthetic data to generate for each, the higher the density the more synthetic points it is going to generate (23). The aim is to shift the learning focus to minority-class points that are harder to learn from based on the high density of majority-class points that are similar to them.

#### 2.4.9 Hybrid techniques

Since it is common to use oversampling algorithms such as SMOTE followed by an undersampling method such as TomekUS and ENNUS (1, 24), two hybrid combinations were investigated in this work: SMOTE with TomekUS (SMOTETomek) and SMOTE with ENNUS (SMOTEENN).

### 2.5 Feature-space transformation: principal component analysis (PCA)

Principal component analysis (PCA) is a method that applies singular value decomposition to multivariate data in order to linearly project it to a lower dimensional space specified by n, in which most of the variance within the data is explained. PCA is widely used for dimensionality reduction in order to compress high-dimensionality data to a lower and potentially more useful representation, allowing for faster and more efficient training of machine-learning models. However, the linearity of this method could hinder its performance when the data of interest exhibits non-linear correlations. To this end, KernelPCA was later proposed as the non-linear form of PCA providing more sophistication to the formation of the principal components (25). Polynomial, radial basis, sigmoid and cosine functions are used as kernels for PCA due to the existence of a dot product space that allows for computing their transformation without explicitly applying the functions, as described in the original paper (25). In this work, PCA and KernelPCA were examined with different numbers of principal components (n=8,16 and 32). Kernels used were 3rd order polynomial function (poly3), radial basis function (rbf), sigmoid function (sig) and cosine function (cos).

### 2.6 Model building: random forest (RF)

Random forest (RF) is one of the most popular machine-learning algorithms due to its robustness and versatility compared to other classical model types. A random forest is composed of an ensemble of decision trees working together to form a model of the training dataset. Each tree in the ensemble is given a different bootstrapped subset of the training set, allowing for variations in the formation of the trees. At inference time, results obtained by each tree are aggregated in order to arrive at a unified prediction (26). In this work, a random forest with 100 trees was used, and each tree was allowed to grow until each leaf node had less than 5 samples, then a split would be prohibited. Having fixed fitting parameters allows for a clearer comparative evaluation of the quality of the class separability in the data given by the different methods, as opposed to tuning those parameters for each case which could cloud the true effects. The implementation provided by Python’s “sklearn.ensemble.RandomForestClassifier” was used.

### 2.7 Evaluation methods

The classification performance was evaluated in two setups where the training-testing split differs:

• Subject-wise splits where the training and testing sets carried data points from different subjects. In this case, two subjects were randomly chosen and their data points were left out for testing, while the remaining 6 subjects’ data points were used for training.
• Section-wise splits where each subject’s data was partitioned into three sections, then randomly choosing one of these sections and leaving it out for testing. This method was illustrated in (15). The partitioning was performed before windowing the data for feature extraction, and the sliding window was only allowed to traverse locally within the sections. This was done to prevent intermediate overlapping windows from leaking information to the testing set.

The class rebalancing methods were only applied to the training set, as they are meant to improve the training process, while testing should strictly include genuine data. In the case of transforming the feature space using PCA or KernelPCA, the training set was used to determine the transformation parameters and the same parameters were then applied to the testing set, in each respective case.

Each examined case was repeated 30 times, with a different random state in each repetition which influences all the steps that require random number generation. This includes the random selection of neighboring pairs in the class rebalancing methods, the random choices in the training-testing splits and the random bootstrapping in the RF model. Therefore, each of the 30 repetitions per case represents a different viewpoint of that particular case. The results from these repetitions are then summarized using the median with its 25th–75th percentiles or the mean with its 95% confidence interval.

The well-known classification evaluation metrics are used in this work: sensitivity, precision, f1-score, accuracy, ROC-AUC (the area under the receiver-operator curve). Those metrics were calculated per class and then averaged, except ROC-AUC which is only concerned with the positive class.

## 3 Results

### 3.1 K value examination

**Figure 1:**
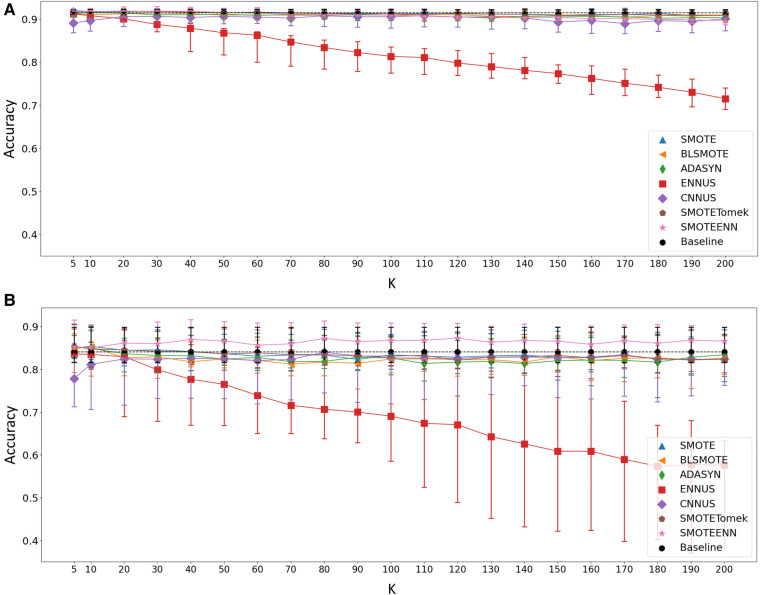
Accuracy of the apnoea classification task after applying the relevant class rebalancing methods as the value of K changes from 5 to 200, in the case of (**A**) section-wise and (**B**) subject-wise split. Baseline represents the performance without applying any class rebalancing method, shown for reference. Shaped points indicate the median value over the 30 repetitions while the bars show the 25th–75th percentile range.

First, the value of K which controls the size of the neighborhood region used in the different neighborhood-based class rebalancing methods was examined. K varied from 5 to 200, and the accuracy of the apnoea classification task was examined when evaluating the models using the testing set under the subject-wise splitting scheme and the section-wise scheme. Those results are shown in Figures 1A,B, respectively. The K parameter influences the working of: SMOTE, BLSMOTE, ADASYN, ENNUS, CNNUS, SMOTETomek and SMOTEENN, and hence, only those methods are shown in the figures.

From the results shown in Figures 1A,B, it was evident that a choice of K=20 was reasonable across all the relevant methods, and this value was used in the remainder of this work.

### 3.2 Class rebalancing methods with feature-space transformation

**Figure 2:**
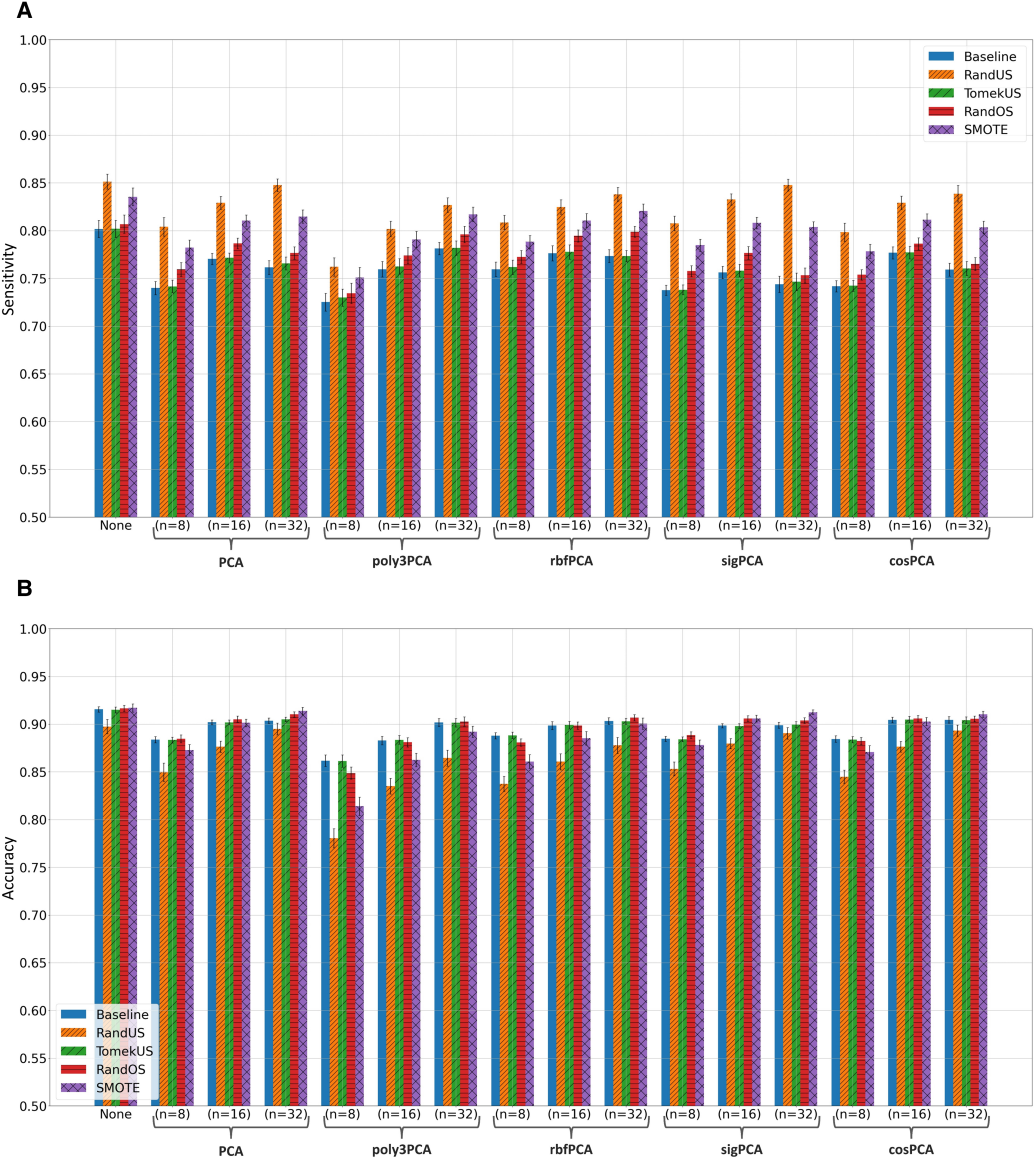
Bar plots of the (**A**) sensitivity and (**B**) accuracy scores achieved when taking the **section-wise** splitting approach in the apnoea classification task. Different colors correspond to different class rebalancing methods, while groups are made based on the feature transformation methods. Bar heights indicate the mean value while the error bars show the 95% confidence interval.

**Figure 3:**
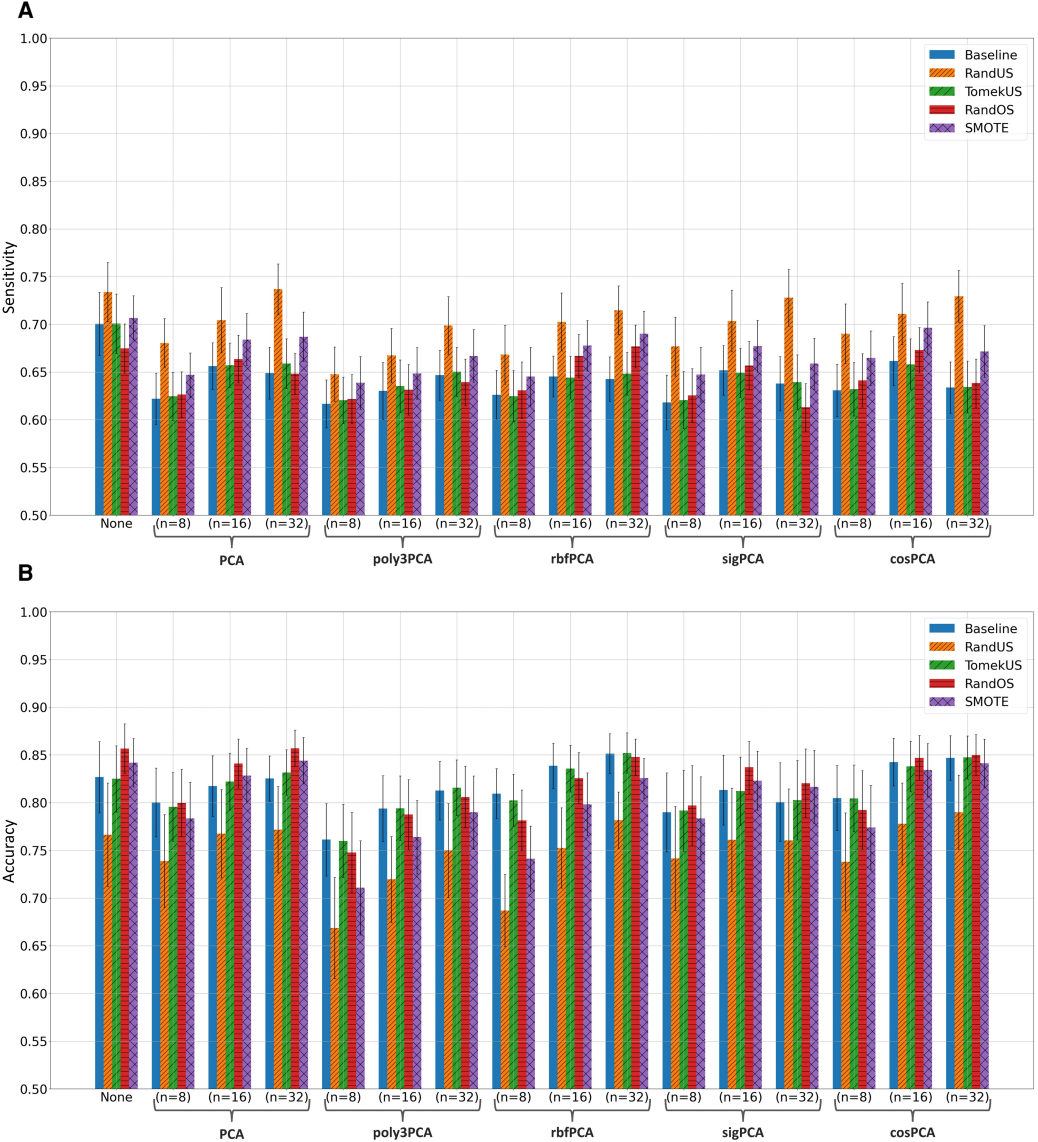
Bar plots of the (**A**) sensitivity and (**B**) accuracy scores achieved when taking the **subject-wise** splitting approach in the apnoea classification task. Different colors correspond to different class rebalancing methods, while groups are made based on the feature transformation methods. Bar heights indicate the mean value while the error bars show the 95% confidence interval.

Fitting the random forest classifier to the training data, without and with feature-space transformation using PCA, poly3PCA, rbfPCA, sigPCA, and cosPCA, each with n=8,16 or 32 was examined and evaluated on their corresponding testing data. Training data was either kept as it is (Baseline) or modified using one of the 10 class rebalancing methods, using K=20 whenever needed. Supplementary Tables S1, S2 list the mean sensitivity, mean precision, mean f1-score, mean accuracy, and mean ROC-AUC scores and their 95% confidence bounds, when evaluated using the section-wise and the subject-wise partitioning, respectively. The highest score (per metric) in each subsection of the tables was written in bold for clarity. After considering the results in Supplementary Tables S1, S2, the top 4 class rebalancing methods (in addition to baseline) which frequently scored highest are visually inspected in bar plots, those methods were: RandUS, TomekUS, RandOS and SMOTE. Figures 2, 3 show their respective sensitivity and accuracy measures in both section-wise and subject-wise splitting approaches, respectively.

Table 4 lists the number of minority-class and majority-class data points in the training set, before and after each class rebalancing method, for the top 4 performing methods as well as the baseline. Those values were averaged over the different transformation approaches to avoid redundancy, as they were found to be identical or very similar across the different feature transformation methods.

**Table 4:** The number of minority-class data points and majority-class data points, total number of data points, and class ratio after applying each of the class rebalancing methods, in both section-wise and subject-wise splitting approaches.

	Method	Minoity-class	Majority-class	Total data points	Class ratio (+:−)
**Section-wise**	Baseline	2,112	9,271	11,383	23:100
	RandUS	2,112	2,112	4,224	1:1
	TomekUS	2,112	9,235	11,347	23:100
	RandOS	9,271	9,271	18,542	1:1
	SMOTE	9,271	9,271	18,542	1:1
**Subject-wise**	Baseline	1,833	8,321	10,154	22:100
	RandUS	1,833	1,833	3,666	1:1
	TomekUS	1,833	8,289	10,122	22:100
	RandOS	8,321	8,321	16,642	1:1
	SMOTE	8,321	8,321	16,642	1:1

## 4 Discussion and conclusion

Inspecting the results shown in Supplementary Tables S1, S2 shows that there were generally 4 class rebalancing methods that often outperformed or were one of the best-performing methods in at least one of the evaluation metrics, across both section-wise and subject-wise splitting schemes. Those were: RandUS, TomekUS, RandOS and SMOTE.

It can also be observed from the Supplementary Tables S1, S2 that undersampling methods (especially RandUS) often outperformed in terms of sensitivity, while they underperformed in terms of accuracy and f1-score. The reduction in accuracy and f1-score could be due to the effect of the amount of data points that were introduced to the RF model during training and how they influenced the growth of the individual trees given the challenging class separability in the data. Undersampling methods reduce the number of data points belonging to the majority class and therefore reduce the total number of data points in the set, consequently reducing the tree depth needed to fit the training data and therefore leading to increased bias and decreased variance. This can be further illustrated by observing Figures 2A, 3A which clearly show that RandUS consistently provided the best sensitivity in each configuration, while Figures 2B, 3B show that oversampling methods were generally providing superior accuracy to RandUS, and comparable accuracy to baseline. However, it is worth noting that TomekUS did not provide significantly different results from baseline. This could be due to the limited effect this method imposed on the training data, as it only removed about 36 data points at most from the majority class, as seen in Table 4.

On the other hand, the figures also show that feature-space transformation did not improve the performance of the methods, and having the original feature-space resulted in higher performance. This is more likely due to the fact that having more features often helps ensemble-based machine-learning models perform better, rather than being due to the quality of the feature-space itself.

**Figure 4:**
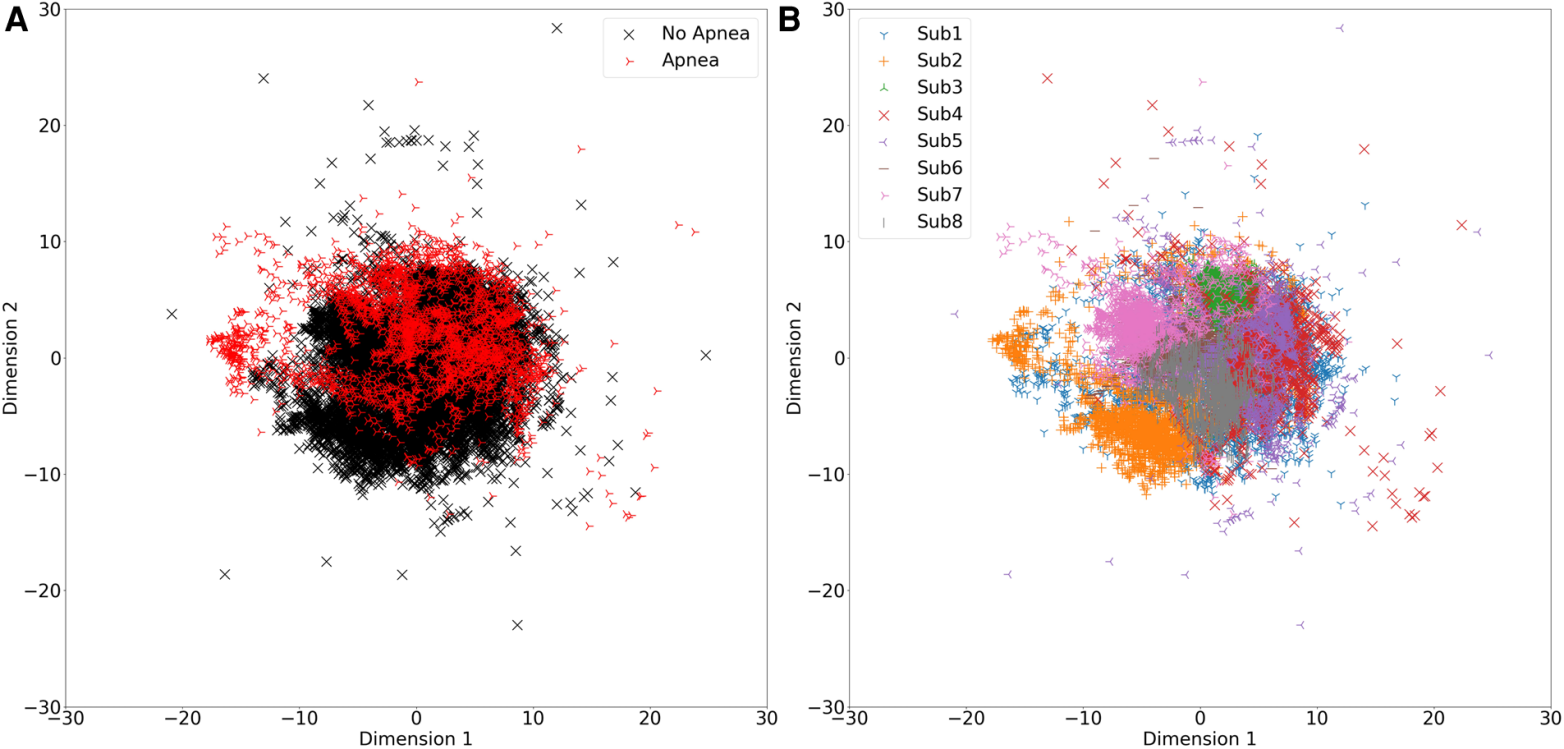
MDS plots showing feature data points in a 2-dimensional space. (**A**) Data points representing apnoea are colored red. (**B**) Data points belonging to different subjects are colored differently, as shown in the corresponding legend.

It is important to note that those sampling-based class rebalancing methods were originally proposed to handle data points that represent independent samples, and which tend to form class-dependant clusters, which is often not the case when handling continuous measurement data where subsets of the data points are drawn from the same entity (subject in the case of this work), causing data dependencies that lead to clusters that are not class-dependant but rather dependant on the entity (subject) they were drawn from. This is illustrated in Figure 4 which shows a multidimensional scaling plot (MDS plot) that allows visualizing high-dimensional feature spaces in 2 dimensions while preserving the relative distances between data points, revealing the significant overlap between apnoea and non-apnoea data points in Figure 4A, and the clear subject-based clustering of data points in Figure 4B. It is likely that the examined feature-space transformations failed to combat this issue.

In conclusion, this work demonstrates the difficulty in handling class imbalance when dealing with physiological data where subject dependencies occur. Although the classification results in this work are unlikely to be generalizable given the limited data, however, the relative comparisons between the performance of the methods amongst themselves and baseline can still be valid when applied to broader datasets. They suggest the use of RandUS if sensitivity is the main concern, which, in this work, achieved 3% to 11% increase in the sensitivity score compared to baseline. Furthermore, artificially augmenting the data in order to increase the overall classification accuracy was shown to be non-trivial, and more methods should be investigated and validated to provide sophisticated alternatives that take subject dependencies into account.

## Data Availability

The raw data supporting the conclusions of this article will be made available by the authors, without undue reservation.
