# Supplementary material for: A comparative study in class imbalance mitigation when working with physiological signals
Source: Front Digit Health. 2024 Mar 26;6:1377165. doi: 10.3389/fdgth.2024.1377165 (PMC11002073; doi:10.3389/fdgth.2024.1377165)
Supplement: Supplementary file 1 [file Table1.pdf]

Frontiers

Supplementary Material

1 SUPPLEMENTARY TABLES AND FIGURES

Table S1: Apnea classification results when applying the class-imbalance treatments in each feature transformation case, and when the RF model was evaluated using the section-wise splitting scheme.

	Treatment	Sensitivity	Precision	F1-Score	Accuracy	ROC-AUC
	**Baseline**	0.8 ± 0.03	$\mathbf{0.9 \pm 0.02}$	0.84 ± 0.02	$\mathbf{0.92 \pm 0.01}$	0.93 ± 0.01
No Transformation	**RandUS**	$\mathbf{0.85 \pm 0.02}$	0.83 ± 0.04	0.84 ± 0.03	0.9 ± 0.02	0.94 ± 0.01
	**TomekUS**	0.8 ± 0.03	$\mathbf{0.9 \pm 0.03}$	0.84 ± 0.02	0.91 ± 0.01	0.93 ± 0.01
	**ENNUS**	0.84 ± 0.02	0.84 ± 0.04	0.84 ± 0.02	0.9 ± 0.02	0.93 ± 0.01
	**CNNUS**	$\mathbf{0.85 \pm 0.02}$	0.83 ± 0.04	0.84 ± 0.03	0.9 ± 0.02	0.93 ± 0.01
	**RandOS**	0.81 ± 0.03	$\mathbf{0.9 \pm 0.02}$	0.84 ± 0.02	$\mathbf{0.92 \pm 0.01}$	0.94 ± 0.01
	**SMOTE**	0.84 ± 0.03	0.88 ± 0.03	$\mathbf{0.85 \pm 0.02}$	$\mathbf{0.92 \pm 0.01}$	0.94 ± 0.01
	**BLSMOTE**	0.84 ± 0.02	0.86 ± 0.04	$\mathbf{0.85 \pm 0.03}$	0.91 ± 0.02	$\mathbf{0.95 \pm 0.01}$
	**ADASYN**	0.84 ± 0.02	0.86 ± 0.04	$\mathbf{0.85 \pm 0.03}$	0.91 ± 0.02	0.94 ± 0.01
	**SMOTETomek**	0.84 ± 0.02	0.88 ± 0.03	$\mathbf{0.85 \pm 0.02}$	$\mathbf{0.92 \pm 0.01}$	0.94 ± 0.01
	**SMOTEENN**	0.82 ± 0.03	0.89 ± 0.03	$\mathbf{0.85 \pm 0.02}$	$\mathbf{0.92 \pm 0.01}$	0.94 ± 0.01
	**Baseline**	0.74 ± 0.02	$\mathbf{0.84 \pm 0.03}$	0.77 ± 0.02	$\mathbf{0.88 \pm 0.01}$	$\mathbf{0.88 \pm 0.02}$
PCA (n = 8)	**RandUS**	$\mathbf{0.8 \pm 0.03}$	0.76 ± 0.04	0.77 ± 0.03	0.85 ± 0.03	$\mathbf{0.88 \pm 0.02}$
	**TomekUS**	0.74 ± 0.02	$\mathbf{0.84 \pm 0.03}$	0.77 ± 0.02	$\mathbf{0.88 \pm 0.01}$	$\mathbf{0.88 \pm 0.02}$
	**ENNUS**	0.78 ± 0.03	0.76 ± 0.05	0.77 ± 0.04	0.85 ± 0.03	$\mathbf{0.88 \pm 0.02}$
	**CNNUS**	0.78 ± 0.02	0.74 ± 0.03	0.75 ± 0.03	0.84 ± 0.02	0.86 ± 0.02
	**RandOS**	0.76 ± 0.02	0.83 ± 0.03	$\mathbf{0.79 \pm 0.02}$	$\mathbf{0.88 \pm 0.01}$	$\mathbf{0.88 \pm 0.02}$
	**SMOTE**	0.78 ± 0.02	0.79 ± 0.03	0.78 ± 0.02	0.87 ± 0.02	$\mathbf{0.88 \pm 0.02}$
	**BLSMOTE**	0.78 ± 0.02	0.76 ± 0.04	0.77 ± 0.03	0.85 ± 0.02	$\mathbf{0.88 \pm 0.02}$
	**ADASYN**	0.79 ± 0.02	0.76 ± 0.03	0.77 ± 0.02	0.86 ± 0.02	$\mathbf{0.88 \pm 0.02}$
	**SMOTETomek**	0.78 ± 0.02	0.79 ± 0.03	$\mathbf{0.79 \pm 0.02}$	0.87 ± 0.02	$\mathbf{0.88 \pm 0.02}$
	**SMOTEENN**	0.76 ± 0.02	0.82 ± 0.03	$\mathbf{0.79 \pm 0.02}$	$\mathbf{0.88 \pm 0.01}$	$\mathbf{0.88 \pm 0.02}$
	**Baseline**	0.77 ± 0.02	$\mathbf{0.88 \pm 0.02}$	0.81 ± 0.01	0.9 ± 0.01	0.91 ± 0.02
PCA (n = 16)	**RandUS**	$\mathbf{0.83 \pm 0.02}$	0.79 ± 0.03	0.81 ± 0.02	0.88 ± 0.02	0.91 ± 0.02
	**TomekUS**	0.77 ± 0.01	$\mathbf{0.88 \pm 0.02}$	0.81 ± 0.01	0.9 ± 0.01	0.91 ± 0.02
	**ENNUS**	0.81 ± 0.02	0.81 ± 0.04	0.81 ± 0.03	0.88 ± 0.02	0.91 ± 0.02
	**CNNUS**	0.81 ± 0.01	0.78 ± 0.02	0.8 ± 0.02	0.87 ± 0.02	0.9 ± 0.01
	**RandOS**	0.79 ± 0.02	$\mathbf{0.88 \pm 0.03}$	0.82 ± 0.02	$\mathbf{0.91 \pm 0.01}$	$\mathbf{0.92 \pm 0.02}$
	**SMOTE**	0.81 ± 0.02	0.85 ± 0.03	$\mathbf{0.83 \pm 0.02}$	0.9 ± 0.01	$\mathbf{0.92 \pm 0.02}$
	**BLSMOTE**	0.81 ± 0.02	0.82 ± 0.03	0.82 ± 0.02	0.89 ± 0.02	$\mathbf{0.92 \pm 0.02}$
	**ADASYN**	0.82 ± 0.02	0.82 ± 0.03	0.82 ± 0.02	0.89 ± 0.02	$\mathbf{0.92 \pm 0.02}$
	**SMOTETomek**	0.81 ± 0.02	0.85 ± 0.03	$\mathbf{0.83 \pm 0.02}$	0.9 ± 0.01	$\mathbf{0.92 \pm 0.02}$

	**SMOTEENN**	0.79 ± 0.02	0.86 ± 0.03	0.82 ± 0.02	0.9 ± 0.01	0.91 ± 0.02
	**Baseline**	0.76 ± 0.02	0.9 ± 0.02	0.81 ± 0.02	0.9 ± 0.01	0.92 ± 0.01
**PCA (n = 32)**	**RandUS**	$\mathbf{0.85 \pm 0.02}$	0.82 ± 0.03	0.83 ± 0.02	0.89 ± 0.02	0.93 ± 0.01
	**TomekUS**	0.77 ± 0.02	0.9 ± 0.02	0.81 ± 0.02	$\mathbf{0.91 \pm 0.01}$	0.92 ± 0.01
	**ENNUS**	0.81 ± 0.02	0.84 ± 0.04	0.82 ± 0.02	0.9 ± 0.02	0.92 ± 0.01
	**CNNUS**	0.83 ± 0.02	0.77 ± 0.03	0.79 ± 0.03	0.86 ± 0.03	0.91 ± 0.02
	**RandOS**	0.78 ± 0.02	$\mathbf{0.91 \pm 0.02}$	0.82 ± 0.02	$\mathbf{0.91 \pm 0.01}$	$\mathbf{0.94 \pm 0.01}$
	**SMOTE**	0.81 ± 0.02	0.88 ± 0.03	$\mathbf{0.84 \pm 0.02}$	$\mathbf{0.91 \pm 0.01}$	$\mathbf{0.94 \pm 0.01}$
	**BLSMOTE**	0.81 ± 0.02	0.86 ± 0.04	0.83 ± 0.03	$\mathbf{0.91 \pm 0.02}$	$\mathbf{0.94 \pm 0.01}$
	**ADASYN**	0.82 ± 0.02	0.86 ± 0.04	$\mathbf{0.84 \pm 0.02}$	$\mathbf{0.91 \pm 0.01}$	$\mathbf{0.94 \pm 0.01}$
	**SMOTETomek**	0.81 ± 0.02	0.88 ± 0.03	$\mathbf{0.84 \pm 0.02}$	$\mathbf{0.91 \pm 0.01}$	$\mathbf{0.94 \pm 0.01}$
	**SMOTEENN**	0.79 ± 0.02	0.89 ± 0.03	0.83 ± 0.02	$\mathbf{0.91 \pm 0.01}$	0.93 ± 0.01
	**Baseline**	0.73 ± 0.03	$\mathbf{0.78 \pm 0.04}$	$\mathbf{0.75 \pm 0.03}$	$\mathbf{0.86 \pm 0.02}$	$\mathbf{0.84 \pm 0.03}$
**Poly3PCA (n = 8)**	**RandUS**	$\mathbf{0.76 \pm 0.03}$	0.69 ± 0.03	0.7 ± 0.03	0.78 ± 0.03	$\mathbf{0.84 \pm 0.03}$
	**TomekUS**	0.73 ± 0.03	$\mathbf{0.78 \pm 0.04}$	$\mathbf{0.75 \pm 0.03}$	$\mathbf{0.86 \pm 0.02}$	$\mathbf{0.84 \pm 0.03}$
	**ENNUS**	$\mathbf{0.76 \pm 0.03}$	0.7 ± 0.04	0.72 ± 0.04	0.8 ± 0.04	$\mathbf{0.84 \pm 0.03}$
	**CNNUS**	0.71 ± 0.02	0.63 ± 0.02	0.62 ± 0.03	0.68 ± 0.04	0.79 ± 0.03
	**RandOS**	0.73 ± 0.03	0.75 ± 0.03	0.74 ± 0.03	0.85 ± 0.02	$\mathbf{0.84 \pm 0.03}$
	**SMOTE**	0.75 ± 0.03	0.71 ± 0.03	0.72 ± 0.03	0.81 ± 0.03	$\mathbf{0.84 \pm 0.03}$
	**BLSMOTE**	0.74 ± 0.03	0.69 ± 0.03	0.71 ± 0.03	0.8 ± 0.03	0.83 ± 0.03
	**ADASYN**	0.75 ± 0.03	0.69 ± 0.03	0.71 ± 0.03	0.8 ± 0.03	$\mathbf{0.84 \pm 0.03}$
	**SMOTETomek**	0.75 ± 0.03	0.71 ± 0.03	0.72 ± 0.03	0.82 ± 0.03	$\mathbf{0.84 \pm 0.03}$
	**SMOTEENN**	0.73 ± 0.03	0.76 ± 0.03	0.74 ± 0.03	0.85 ± 0.02	$\mathbf{0.84 \pm 0.03}$
	**Baseline**	0.76 ± 0.02	$\mathbf{0.82 \pm 0.03}$	0.78 ± 0.02	$\mathbf{0.88 \pm 0.01}$	$\mathbf{0.88 \pm 0.02}$
**Poly3PCA (n = 16)**	**RandUS**	$\mathbf{0.8 \pm 0.02}$	0.74 ± 0.03	0.76 ± 0.03	0.84 ± 0.02	$\mathbf{0.88 \pm 0.02}$
	**TomekUS**	0.76 ± 0.02	$\mathbf{0.82 \pm 0.03}$	$\mathbf{0.79 \pm 0.02}$	$\mathbf{0.88 \pm 0.01}$	$\mathbf{0.88 \pm 0.02}$
	**ENNUS**	0.79 ± 0.03	0.75 ± 0.04	0.76 ± 0.04	0.84 ± 0.03	$\mathbf{0.88 \pm 0.03}$
	**CNNUS**	0.76 ± 0.02	0.69 ± 0.02	0.71 ± 0.03	0.79 ± 0.03	0.85 ± 0.02
	**RandOS**	0.77 ± 0.02	0.81 ± 0.03	$\mathbf{0.79 \pm 0.02}$	$\mathbf{0.88 \pm 0.01}$	$\mathbf{0.88 \pm 0.02}$
	**SMOTE**	0.79 ± 0.02	0.77 ± 0.03	0.78 ± 0.03	0.86 ± 0.02	$\mathbf{0.88 \pm 0.02}$
	**BLSMOTE**	0.79 ± 0.03	0.75 ± 0.04	0.77 ± 0.03	0.85 ± 0.03	$\mathbf{0.88 \pm 0.02}$
	**ADASYN**	0.79 ± 0.02	0.75 ± 0.04	0.77 ± 0.03	0.85 ± 0.03	$\mathbf{0.88 \pm 0.02}$
	**SMOTETomek**	0.79 ± 0.02	0.77 ± 0.03	0.78 ± 0.03	0.86 ± 0.02	$\mathbf{0.88 \pm 0.02}$
	**SMOTEENN**	0.77 ± 0.02	0.81 ± 0.03	$\mathbf{0.79 \pm 0.02}$	$\mathbf{0.88 \pm 0.01}$	0.87 ± 0.02
	**Baseline**	0.78 ± 0.02	$\mathbf{0.87 \pm 0.03}$	0.81 ± 0.02	$\mathbf{0.9 \pm 0.01}$	$\mathbf{0.91 \pm 0.02}$
**Poly3PCA (n = 32)**	**RandUS**	$\mathbf{0.83 \pm 0.02}$	0.78 ± 0.03	0.79 ± 0.03	0.86 ± 0.02	$\mathbf{0.91 \pm 0.02}$
	**TomekUS**	0.78 ± 0.02	$\mathbf{0.87 \pm 0.03}$	0.81 ± 0.02	$\mathbf{0.9 \pm 0.01}$	0.9 ± 0.02
	**ENNUS**	0.82 ± 0.02	0.79 ± 0.04	0.8 ± 0.03	0.87 ± 0.03	$\mathbf{0.91 \pm 0.02}$
	**CNNUS**	0.81 ± 0.02	0.76 ± 0.03	0.78 ± 0.03	0.85 ± 0.03	0.89 ± 0.02
	**RandOS**	0.8 ± 0.02	0.86 ± 0.03	$\mathbf{0.82 \pm 0.02}$	$\mathbf{0.9 \pm 0.01}$	$\mathbf{0.91 \pm 0.02}$
	**SMOTE**	0.82 ± 0.02	0.82 ± 0.03	$\mathbf{0.82 \pm 0.02}$	0.89 ± 0.02	$\mathbf{0.91 \pm 0.02}$
	**BLSMOTE**	0.82 ± 0.02	0.8 ± 0.04	0.81 ± 0.03	0.88 ± 0.02	$\mathbf{0.91 \pm 0.02}$
	**ADASYN**	0.82 ± 0.02	0.8 ± 0.04	0.81 ± 0.03	0.88 ± 0.02	$\mathbf{0.91 \pm 0.02}$

	**SMOTETomek**	0.82 ± 0.02	0.82 ± 0.03	$\mathbf{0.82 \pm 0.02}$	0.89 ± 0.02	$\mathbf{0.91 \pm 0.02}$
	**SMOTEENN**	0.8 ± 0.02	0.85 ± 0.03	$\mathbf{0.82 \pm 0.02}$	$\mathbf{0.9 \pm 0.01}$	$\mathbf{0.91 \pm 0.02}$
	**Baseline**	0.76 ± 0.02	$\mathbf{0.84 \pm 0.03}$	$\mathbf{0.79 \pm 0.02}$	$\mathbf{0.89 \pm 0.01}$	$\mathbf{0.89 \pm 0.02}$
**rbfPCA (n = 8)**	**RandUS**	$\mathbf{0.81 \pm 0.02}$	0.74 ± 0.03	0.76 ± 0.03	0.84 ± 0.02	$\mathbf{0.89 \pm 0.02}$
	**TomekUS**	0.76 ± 0.02	$\mathbf{0.84 \pm 0.03}$	$\mathbf{0.79 \pm 0.02}$	$\mathbf{0.89 \pm 0.01}$	$\mathbf{0.89 \pm 0.02}$
	**ENNUS**	0.8 ± 0.02	0.76 ± 0.03	0.77 ± 0.03	0.85 ± 0.03	$\mathbf{0.89 \pm 0.02}$
	**CNNUS**	0.78 ± 0.02	0.72 ± 0.02	0.74 ± 0.02	0.82 ± 0.02	0.86 ± 0.02
	**RandOS**	0.77 ± 0.02	0.81 ± 0.03	$\mathbf{0.79 \pm 0.02}$	0.88 ± 0.01	$\mathbf{0.89 \pm 0.02}$
	**SMOTE**	0.79 ± 0.02	0.77 ± 0.03	0.78 ± 0.02	0.86 ± 0.02	$\mathbf{0.89 \pm 0.02}$
	**BLSMOTE**	0.79 ± 0.02	0.75 ± 0.04	0.76 ± 0.03	0.84 ± 0.03	0.88 ± 0.02
	**ADASYN**	0.79 ± 0.02	0.75 ± 0.03	0.76 ± 0.03	0.84 ± 0.03	$\mathbf{0.89 \pm 0.02}$
	**SMOTETomek**	0.79 ± 0.02	0.77 ± 0.03	0.78 ± 0.02	0.86 ± 0.02	$\mathbf{0.89 \pm 0.02}$
	**SMOTEENN**	0.77 ± 0.02	0.82 ± 0.03	$\mathbf{0.79 \pm 0.02}$	0.88 ± 0.01	$\mathbf{0.89 \pm 0.02}$
	**Baseline**	0.78 ± 0.02	$\mathbf{0.86 \pm 0.03}$	0.81 ± 0.02	$\mathbf{0.9 \pm 0.01}$	$\mathbf{0.91 \pm 0.02}$
**rbfPCA (n = 16)**	**RandUS**	$\mathbf{0.82 \pm 0.02}$	0.77 ± 0.03	0.79 ± 0.03	0.86 ± 0.02	$\mathbf{0.91 \pm 0.02}$
	**TomekUS**	0.78 ± 0.02	$\mathbf{0.86 \pm 0.03}$	0.81 ± 0.02	$\mathbf{0.9 \pm 0.01}$	$\mathbf{0.91 \pm 0.02}$
	**ENNUS**	0.81 ± 0.03	0.79 ± 0.04	0.8 ± 0.04	0.87 ± 0.03	$\mathbf{0.91 \pm 0.02}$
	**CNNUS**	0.81 ± 0.02	0.75 ± 0.03	0.77 ± 0.03	0.84 ± 0.03	0.89 ± 0.02
	**RandOS**	0.79 ± 0.02	0.85 ± 0.03	$\mathbf{0.82 \pm 0.02}$	$\mathbf{0.9 \pm 0.01}$	$\mathbf{0.91 \pm 0.02}$
	**SMOTE**	0.81 ± 0.02	0.81 ± 0.04	0.81 ± 0.03	0.89 ± 0.02	$\mathbf{0.91 \pm 0.02}$
	**BLSMOTE**	0.81 ± 0.02	0.79 ± 0.04	0.8 ± 0.03	0.87 ± 0.02	$\mathbf{0.91 \pm 0.02}$
	**ADASYN**	0.81 ± 0.02	0.79 ± 0.04	0.8 ± 0.03	0.87 ± 0.02	$\mathbf{0.91 \pm 0.02}$
	**SMOTETomek**	0.81 ± 0.02	0.81 ± 0.04	0.81 ± 0.03	0.89 ± 0.02	$\mathbf{0.91 \pm 0.02}$
	**SMOTEENN**	0.8 ± 0.02	0.84 ± 0.03	$\mathbf{0.82 \pm 0.02}$	$\mathbf{0.9 \pm 0.01}$	0.9 ± 0.02
	**Baseline**	0.77 ± 0.02	$\mathbf{0.88 \pm 0.03}$	0.81 ± 0.02	0.9 ± 0.01	0.92 ± 0.02
**rbfPCA (n = 32)**	**RandUS**	$\mathbf{0.84 \pm 0.02}$	0.79 ± 0.03	0.81 ± 0.03	0.88 ± 0.02	0.92 ± 0.02
	**TomekUS**	0.77 ± 0.02	$\mathbf{0.88 \pm 0.03}$	0.81 ± 0.02	0.9 ± 0.01	0.92 ± 0.02
	**ENNUS**	0.82 ± 0.02	0.82 ± 0.04	0.82 ± 0.03	0.89 ± 0.02	0.92 ± 0.02
	**CNNUS**	0.83 ± 0.02	0.77 ± 0.03	0.79 ± 0.03	0.86 ± 0.03	0.9 ± 0.02
	**RandOS**	0.8 ± 0.02	0.87 ± 0.03	$\mathbf{0.83 \pm 0.02}$	$\mathbf{0.91 \pm 0.01}$	$\mathbf{0.93 \pm 0.02}$
	**SMOTE**	0.82 ± 0.02	0.84 ± 0.04	$\mathbf{0.83 \pm 0.03}$	0.9 ± 0.02	$\mathbf{0.93 \pm 0.02}$
	**BLSMOTE**	0.82 ± 0.02	0.82 ± 0.04	0.82 ± 0.03	0.89 ± 0.02	$\mathbf{0.93 \pm 0.02}$
	**ADASYN**	0.83 ± 0.02	0.82 ± 0.04	0.82 ± 0.03	0.89 ± 0.02	$\mathbf{0.93 \pm 0.02}$
	**SMOTETomek**	0.82 ± 0.02	0.84 ± 0.04	$\mathbf{0.83 \pm 0.02}$	0.9 ± 0.02	$\mathbf{0.93 \pm 0.02}$
	**SMOTEENN**	0.8 ± 0.02	0.87 ± 0.03	$\mathbf{0.83 \pm 0.02}$	$\mathbf{0.91 \pm 0.01}$	0.92 ± 0.02
	**Baseline**	0.74 ± 0.01	$\mathbf{0.84 \pm 0.03}$	0.77 ± 0.01	0.88 ± 0.01	0.88 ± 0.02
**sigPCA (n = 8)**	**RandUS**	$\mathbf{0.81 \pm 0.02}$	0.76 ± 0.03	0.78 ± 0.03	0.85 ± 0.02	$\mathbf{0.89 \pm 0.02}$
	**TomekUS**	0.74 ± 0.01	$\mathbf{0.84 \pm 0.03}$	0.77 ± 0.01	0.88 ± 0.01	0.88 ± 0.02
	**ENNUS**	0.78 ± 0.02	0.78 ± 0.04	0.78 ± 0.03	0.87 ± 0.02	0.88 ± 0.02
	**CNNUS**	0.77 ± 0.02	0.69 ± 0.02	0.71 ± 0.02	0.78 ± 0.03	0.85 ± 0.02
	**RandOS**	0.76 ± 0.02	$\mathbf{0.84 \pm 0.03}$	$\mathbf{0.79 \pm 0.02}$	$\mathbf{0.89 \pm 0.01}$	$\mathbf{0.89 \pm 0.02}$
	**SMOTE**	0.78 ± 0.02	0.8 ± 0.03	$\mathbf{0.79 \pm 0.02}$	0.88 ± 0.01	$\mathbf{0.89 \pm 0.02}$
	**BLSMOTE**	0.78 ± 0.02	0.78 ± 0.04	0.78 ± 0.03	0.87 ± 0.02	0.88 ± 0.02

	**ADASYN**	0.79 ± 0.02	0.78 ± 0.04	0.78 ± 0.03	0.87 ± 0.02	0.88 ± 0.02
	**SMOTETomek**	0.78 ± 0.02	0.8 ± 0.03	**0.79 ± 0.02**	0.88 ± 0.01	**0.89 ± 0.02**
	**SMOTEENN**	0.76 ± 0.02	0.82 ± 0.03	**0.79 ± 0.02**	0.88 ± 0.01	**0.89 ± 0.02**
	**Baseline**	0.76 ± 0.02	0.88 ± 0.02	0.8 ± 0.01	0.9 ± 0.01	0.9 ± 0.02
**sigPCA (n = 16)**	**RandUS**	**0.83 ± 0.02**	0.8 ± 0.03	0.81 ± 0.02	0.88 ± 0.01	0.91 ± 0.02
	**TomekUS**	0.76 ± 0.02	0.88 ± 0.02	0.8 ± 0.02	0.9 ± 0.01	0.9 ± 0.02
	**ENNUS**	0.8 ± 0.01	0.82 ± 0.03	0.81 ± 0.02	0.89 ± 0.01	0.9 ± 0.02
	**CNNUS**	0.81 ± 0.02	0.73 ± 0.03	0.75 ± 0.03	0.82 ± 0.03	0.89 ± 0.02
	**RandOS**	0.78 ± 0.02	**0.89 ± 0.03**	0.82 ± 0.02	**0.91 ± 0.01**	0.91 ± 0.02
	**SMOTE**	0.81 ± 0.02	0.86 ± 0.03	**0.83 ± 0.02**	**0.91 ± 0.01**	**0.92 ± 0.02**
	**BLSMOTE**	0.81 ± 0.02	0.85 ± 0.02	0.82 ± 0.02	0.9 ± 0.01	**0.92 ± 0.02**
	**ADASYN**	0.81 ± 0.02	0.84 ± 0.03	**0.83 ± 0.02**	0.9 ± 0.01	**0.92 ± 0.02**
	**SMOTETomek**	0.81 ± 0.02	0.86 ± 0.03	**0.83 ± 0.02**	**0.91 ± 0.01**	**0.92 ± 0.02**
	**SMOTEENN**	0.79 ± 0.02	0.88 ± 0.02	**0.83 ± 0.02**	**0.91 ± 0.01**	0.91 ± 0.02
	**Baseline**	0.74 ± 0.02	0.9 ± 0.01	0.79 ± 0.02	0.9 ± 0.01	0.91 ± 0.02
**sigPCA (n = 32)**	**RandUS**	**0.85 ± 0.02**	0.81 ± 0.03	0.83 ± 0.02	0.89 ± 0.02	**0.93 ± 0.01**
	**TomekUS**	0.75 ± 0.03	0.9 ± 0.02	0.79 ± 0.02	0.9 ± 0.01	0.91 ± 0.02
	**ENNUS**	0.79 ± 0.02	0.85 ± 0.04	0.81 ± 0.02	0.9 ± 0.01	0.92 ± 0.01
	**CNNUS**	0.81 ± 0.02	0.71 ± 0.03	0.72 ± 0.04	0.79 ± 0.04	0.9 ± 0.02
	**RandOS**	0.75 ± 0.02	**0.91 ± 0.01**	0.8 ± 0.02	0.9 ± 0.01	**0.93 ± 0.01**
	**SMOTE**	0.8 ± 0.02	0.89 ± 0.02	**0.84 ± 0.01**	**0.91 ± 0.01**	**0.93 ± 0.01**
	**BLSMOTE**	0.79 ± 0.02	0.87 ± 0.03	0.82 ± 0.02	**0.91 ± 0.01**	**0.93 ± 0.01**
	**ADASYN**	0.8 ± 0.02	0.87 ± 0.03	0.83 ± 0.02	**0.91 ± 0.01**	**0.93 ± 0.01**
	**SMOTETomek**	0.8 ± 0.02	0.89 ± 0.02	**0.84 ± 0.02**	**0.91 ± 0.01**	**0.93 ± 0.01**
	**SMOTEENN**	0.79 ± 0.02	0.9 ± 0.02	0.83 ± 0.02	**0.91 ± 0.01**	**0.93 ± 0.01**
	**Baseline**	0.74 ± 0.02	**0.84 ± 0.03**	**0.78 ± 0.02**	**0.88 ± 0.01**	**0.88 ± 0.02**
**cosPCA (n = 8)**	**RandUS**	**0.8 ± 0.03**	0.75 ± 0.03	0.77 ± 0.02	0.84 ± 0.02	**0.88 ± 0.02**
	**TomekUS**	0.74 ± 0.02	**0.84 ± 0.03**	**0.78 ± 0.02**	**0.88 ± 0.01**	**0.88 ± 0.02**
	**ENNUS**	0.79 ± 0.02	0.76 ± 0.03	0.77 ± 0.03	0.86 ± 0.02	**0.88 ± 0.02**
	**CNNUS**	0.77 ± 0.02	0.7 ± 0.02	0.72 ± 0.02	0.8 ± 0.02	0.85 ± 0.02
	**RandOS**	0.75 ± 0.02	0.82 ± 0.03	**0.78 ± 0.02**	**0.88 ± 0.01**	**0.88 ± 0.02**
	**SMOTE**	0.78 ± 0.02	0.79 ± 0.04	**0.78 ± 0.02**	0.87 ± 0.02	**0.88 ± 0.02**
	**BLSMOTE**	0.78 ± 0.02	0.77 ± 0.04	0.77 ± 0.03	0.86 ± 0.02	**0.88 ± 0.02**
	**ADASYN**	0.78 ± 0.02	0.77 ± 0.04	0.77 ± 0.03	0.86 ± 0.02	**0.88 ± 0.02**
	**SMOTETomek**	0.78 ± 0.02	0.79 ± 0.04	**0.78 ± 0.02**	0.87 ± 0.02	**0.88 ± 0.02**
	**SMOTEENN**	0.76 ± 0.02	0.82 ± 0.03	**0.78 ± 0.02**	**0.88 ± 0.01**	**0.88 ± 0.02**
	**Baseline**	0.78 ± 0.02	**0.88 ± 0.03**	0.82 ± 0.02	0.9 ± 0.01	0.91 ± 0.02
**cosPCA (n = 16)**	**RandUS**	**0.83 ± 0.02**	0.79 ± 0.03	0.81 ± 0.02	0.88 ± 0.02	0.91 ± 0.02
	**TomekUS**	0.78 ± 0.02	**0.88 ± 0.03**	0.82 ± 0.02	0.9 ± 0.01	0.91 ± 0.02
	**ENNUS**	0.81 ± 0.02	0.82 ± 0.03	0.81 ± 0.02	0.89 ± 0.02	0.91 ± 0.02
	**CNNUS**	0.81 ± 0.02	0.75 ± 0.02	0.77 ± 0.02	0.84 ± 0.02	0.89 ± 0.02
	**RandOS**	0.79 ± 0.02	**0.88 ± 0.03**	0.82 ± 0.02	**0.91 ± 0.01**	0.91 ± 0.02
	**SMOTE**	0.81 ± 0.02	0.85 ± 0.03	**0.83 ± 0.02**	0.9 ± 0.01	**0.92 ± 0.02**

	**BLSMOTE**	0.81 ± 0.02	0.84 ± 0.03	0.82 ± 0.02	0.9 ± 0.02	$\mathbf{0.92 \pm 0.02}$
	**ADASYN**	0.82 ± 0.02	0.83 ± 0.04	0.82 ± 0.02	0.9 ± 0.02	$\mathbf{0.92 \pm 0.02}$
	**SMOTETomek**	0.81 ± 0.02	0.85 ± 0.03	$\mathbf{0.83 \pm 0.02}$	0.9 ± 0.01	$\mathbf{0.92 \pm 0.02}$
	**SMOTEENN**	0.8 ± 0.02	0.87 ± 0.03	0.82 ± 0.02	0.9 ± 0.01	0.91 ± 0.02
	**Baseline**	0.76 ± 0.02	$\mathbf{0.91 \pm 0.02}$	0.81 ± 0.02	0.9 ± 0.01	0.92 ± 0.01
**cosPCA (n = 32)**	**RandUS**	$\mathbf{0.84 \pm 0.02}$	0.82 ± 0.03	$\mathbf{0.83 \pm 0.03}$	0.89 ± 0.02	$\mathbf{0.93 \pm 0.01}$
	**TomekUS**	0.76 ± 0.02	0.9 ± 0.02	0.81 ± 0.02	0.9 ± 0.01	0.92 ± 0.01
	**ENNUS**	0.8 ± 0.03	0.85 ± 0.04	0.82 ± 0.03	0.9 ± 0.01	0.92 ± 0.01
	**CNNUS**	0.82 ± 0.02	0.74 ± 0.03	0.76 ± 0.04	0.82 ± 0.04	0.91 ± 0.02
	**RandOS**	0.77 ± 0.02	0.9 ± 0.02	0.81 ± 0.02	$\mathbf{0.91 \pm 0.01}$	$\mathbf{0.93 \pm 0.01}$
	**SMOTE**	0.8 ± 0.02	0.88 ± 0.02	$\mathbf{0.83 \pm 0.02}$	$\mathbf{0.91 \pm 0.01}$	$\mathbf{0.93 \pm 0.01}$
	**BLSMOTE**	0.8 ± 0.02	0.87 ± 0.03	$\mathbf{0.83 \pm 0.02}$	$\mathbf{0.91 \pm 0.01}$	$\mathbf{0.93 \pm 0.01}$
	**ADASYN**	0.81 ± 0.02	0.87 ± 0.02	$\mathbf{0.83 \pm 0.02}$	$\mathbf{0.91 \pm 0.01}$	$\mathbf{0.93 \pm 0.01}$
	**SMOTETomek**	0.8 ± 0.02	0.88 ± 0.02	$\mathbf{0.83 \pm 0.02}$	$\mathbf{0.91 \pm 0.01}$	$\mathbf{0.93 \pm 0.01}$
	**SMOTEENN**	0.79 ± 0.02	0.89 ± 0.02	0.82 ± 0.02	$\mathbf{0.91 \pm 0.01}$	$\mathbf{0.93 \pm 0.01}$

Table S2: Apnea classification results when applying the class-imbalance treatments in each feature transformation case, and when the RF model was evaluated using the subject-wise splitting scheme.

	Treatment	Sensitivity	Precision	F1-Score	Accuracy	ROC-AUC
	**Baseline**	0.7 ± 0.09	0.75 ± 0.13	$\mathbf{0.7 \pm 0.12}$	0.83 ± 0.1	0.82 ± 0.1
**No Transformation**	**RandUS**	$\mathbf{0.73 \pm 0.09}$	0.7 ± 0.12	0.67 ± 0.15	0.77 ± 0.15	0.83 ± 0.1
	**TomekUS**	0.7 ± 0.09	0.75 ± 0.12	0.69 ± 0.11	0.82 ± 0.1	0.82 ± 0.1
	**ENNUS**	$\mathbf{0.73 \pm 0.08}$	0.71 ± 0.12	0.69 ± 0.14	0.78 ± 0.14	0.83 ± 0.09
	**CNNUS**	0.72 ± 0.08	0.69 ± 0.11	0.66 ± 0.14	0.76 ± 0.14	0.81 ± 0.09
	**RandOS**	0.67 ± 0.07	$\mathbf{0.79 \pm 0.11}$	0.69 ± 0.09	$\mathbf{0.86 \pm 0.07}$	0.83 ± 0.08
	**SMOTE**	0.71 ± 0.06	0.74 ± 0.11	$\mathbf{0.7 \pm 0.09}$	0.84 ± 0.07	0.83 ± 0.08
	**BLSMOTE**	0.69 ± 0.08	0.72 ± 0.13	0.67 ± 0.12	0.8 ± 0.12	0.82 ± 0.1
	**ADASYN**	0.7 ± 0.08	0.72 ± 0.12	0.68 ± 0.11	0.81 ± 0.1	0.83 ± 0.1
	**SMOTETomek**	0.7 ± 0.07	0.74 ± 0.11	$\mathbf{0.7 \pm 0.1}$	0.84 ± 0.08	$\mathbf{0.84 \pm 0.08}$
	**SMOTEENN**	0.69 ± 0.07	0.77 ± 0.11	$\mathbf{0.7 \pm 0.09}$	0.85 ± 0.07	$\mathbf{0.84 \pm 0.09}$
	**Baseline**	0.62 ± 0.07	$\mathbf{0.7 \pm 0.12}$	0.62 ± 0.1	$\mathbf{0.8 \pm 0.1}$	0.74 ± 0.11
**PCA (n = 8)**	**RandUS**	$\mathbf{0.68 \pm 0.07}$	0.66 ± 0.11	$\mathbf{0.63 \pm 0.13}$	0.74 ± 0.14	$\mathbf{0.76 \pm 0.1}$
	**TomekUS**	0.62 ± 0.07	$\mathbf{0.7 \pm 0.12}$	0.62 ± 0.1	$\mathbf{0.8 \pm 0.1}$	0.74 ± 0.1
	**ENNUS**	$\mathbf{0.68 \pm 0.06}$	0.68 ± 0.11	$\mathbf{0.63 \pm 0.11}$	0.75 ± 0.13	$\mathbf{0.76 \pm 0.09}$
	**CNNUS**	0.65 ± 0.07	0.65 ± 0.12	0.61 ± 0.12	0.73 ± 0.13	0.73 ± 0.11
	**RandOS**	0.63 ± 0.07	$\mathbf{0.7 \pm 0.11}$	0.62 ± 0.09	$\mathbf{0.8 \pm 0.1}$	0.75 ± 0.1
	**SMOTE**	0.65 ± 0.06	0.68 ± 0.11	$\mathbf{0.63 \pm 0.1}$	0.78 ± 0.11	0.75 ± 0.1
	**BLSMOTE**	0.65 ± 0.07	0.67 ± 0.11	0.62 ± 0.1	0.77 ± 0.11	0.75 ± 0.09
	**ADASYN**	0.65 ± 0.07	0.67 ± 0.11	0.62 ± 0.11	0.77 ± 0.11	0.75 ± 0.1
	**SMOTETomek**	0.65 ± 0.06	0.68 ± 0.11	$\mathbf{0.63 \pm 0.09}$	0.79 ± 0.1	0.75 ± 0.1

	**SMOTEENN**	0.63 ± 0.07	$\mathbf{0.7 \pm 0.11}$	0.62 ± 0.09	$\mathbf{0.8 \pm 0.1}$	0.75 ± 0.1
	**Baseline**	0.66 ± 0.07	0.73 ± 0.12	0.66 ± 0.1	0.82 ± 0.09	0.77 ± 0.09
**PCA (n = 16)**	**RandUS**	$\mathbf{0.7 \pm 0.1}$	0.68 ± 0.11	0.66 ± 0.13	0.77 ± 0.13	0.79 ± 0.09
	**TomekUS**	0.66 ± 0.07	0.73 ± 0.11	0.66 ± 0.09	0.82 ± 0.08	0.77 ± 0.09
	**ENNUS**	0.69 ± 0.08	0.68 ± 0.12	0.65 ± 0.12	0.77 ± 0.12	0.78 ± 0.09
	**CNNUS**	$\mathbf{0.7 \pm 0.07}$	0.67 ± 0.1	0.65 ± 0.1	0.77 ± 0.11	0.78 ± 0.08
	**RandOS**	0.66 ± 0.07	$\mathbf{0.76 \pm 0.11}$	0.67 ± 0.1	$\mathbf{0.84 \pm 0.07}$	0.79 ± 0.08
	**SMOTE**	0.68 ± 0.08	0.73 ± 0.11	$\mathbf{0.68 \pm 0.1}$	0.83 ± 0.08	0.79 ± 0.08
	**BLSMOTE**	0.69 ± 0.07	0.71 ± 0.11	0.67 ± 0.1	0.82 ± 0.07	$\mathbf{0.8 \pm 0.07}$
	**ADASYN**	0.69 ± 0.08	0.71 ± 0.1	0.67 ± 0.1	0.82 ± 0.08	0.79 ± 0.07
	**SMOTETomek**	0.68 ± 0.08	0.73 ± 0.11	$\mathbf{0.68 \pm 0.1}$	0.83 ± 0.07	0.79 ± 0.08
	**SMOTEENN**	0.67 ± 0.07	0.75 ± 0.11	$\mathbf{0.68 \pm 0.09}$	$\mathbf{0.84 \pm 0.07}$	0.78 ± 0.09
	**Baseline**	0.65 ± 0.08	0.72 ± 0.1	0.65 ± 0.08	0.83 ± 0.07	0.8 ± 0.09
**PCA (n = 32)**	**RandUS**	$\mathbf{0.74 \pm 0.07}$	0.69 ± 0.11	0.68 ± 0.13	0.77 ± 0.13	$\mathbf{0.83 \pm 0.08}$
	**TomekUS**	0.66 ± 0.07	0.73 ± 0.11	0.66 ± 0.09	0.83 ± 0.07	0.8 ± 0.09
	**ENNUS**	0.7 ± 0.07	0.7 ± 0.11	0.67 ± 0.11	0.79 ± 0.11	0.81 ± 0.09
	**CNNUS**	0.71 ± 0.07	0.65 ± 0.08	0.63 ± 0.12	0.72 ± 0.14	0.81 ± 0.08
	**RandOS**	0.65 ± 0.06	$\mathbf{0.79 \pm 0.11}$	0.67 ± 0.08	$\mathbf{0.86 \pm 0.05}$	$\mathbf{0.83 \pm 0.07}$
	**SMOTE**	0.69 ± 0.07	0.75 ± 0.12	$\mathbf{0.69 \pm 0.1}$	0.84 ± 0.07	$\mathbf{0.83 \pm 0.08}$
	**BLSMOTE**	0.68 ± 0.07	0.74 ± 0.12	0.67 ± 0.09	0.84 ± 0.05	$\mathbf{0.83 \pm 0.07}$
	**ADASYN**	0.7 ± 0.07	0.73 ± 0.11	0.68 ± 0.1	0.83 ± 0.06	$\mathbf{0.83 \pm 0.07}$
	**SMOTETomek**	0.69 ± 0.06	0.75 ± 0.11	$\mathbf{0.69 \pm 0.09}$	0.85 ± 0.06	$\mathbf{0.83 \pm 0.07}$
	**SMOTEENN**	0.67 ± 0.07	0.77 ± 0.11	0.68 ± 0.08	$\mathbf{0.86 \pm 0.05}$	$\mathbf{0.83 \pm 0.07}$
	**Baseline**	0.62 ± 0.07	$\mathbf{0.66 \pm 0.12}$	$\mathbf{0.6 \pm 0.1}$	$\mathbf{0.76 \pm 0.11}$	0.68 ± 0.08
**Poly3PCA (n = 8)**	**RandUS**	$\mathbf{0.65 \pm 0.08}$	0.62 ± 0.09	0.58 ± 0.13	0.67 ± 0.15	0.68 ± 0.1
	**TomekUS**	0.62 ± 0.07	$\mathbf{0.66 \pm 0.12}$	$\mathbf{0.6 \pm 0.1}$	$\mathbf{0.76 \pm 0.11}$	0.68 ± 0.08
	**ENNUS**	$\mathbf{0.65 \pm 0.07}$	0.63 ± 0.1	0.58 ± 0.13	0.68 ± 0.15	$\mathbf{0.69 \pm 0.09}$
	**CNNUS**	0.62 ± 0.08	0.59 ± 0.07	0.55 ± 0.13	0.64 ± 0.15	0.64 ± 0.09
	**RandOS**	0.62 ± 0.07	$\mathbf{0.66 \pm 0.12}$	$\mathbf{0.6 \pm 0.11}$	0.75 ± 0.12	0.68 ± 0.08
	**SMOTE**	0.64 ± 0.08	0.64 ± 0.11	0.59 ± 0.12	0.71 ± 0.14	0.68 ± 0.09
	**BLSMOTE**	0.64 ± 0.07	0.63 ± 0.1	0.59 ± 0.11	0.71 ± 0.12	0.68 ± 0.08
	**ADASYN**	0.64 ± 0.07	0.63 ± 0.1	0.59 ± 0.12	0.7 ± 0.13	0.68 ± 0.08
	**SMOTETomek**	0.64 ± 0.07	0.63 ± 0.1	0.59 ± 0.12	0.71 ± 0.13	0.68 ± 0.09
	**SMOTEENN**	0.61 ± 0.08	0.64 ± 0.11	0.59 ± 0.11	0.75 ± 0.12	0.65 ± 0.09
	**Baseline**	0.63 ± 0.08	$\mathbf{0.69 \pm 0.12}$	$\mathbf{0.63 \pm 0.1}$	$\mathbf{0.79 \pm 0.1}$	0.71 ± 0.1
**Poly3PCA (n = 16)**	**RandUS**	$\mathbf{0.67 \pm 0.08}$	0.64 ± 0.09	0.61 ± 0.12	0.72 ± 0.13	0.71 ± 0.1
	**TomekUS**	0.64 ± 0.08	$\mathbf{0.69 \pm 0.12}$	$\mathbf{0.63 \pm 0.1}$	$\mathbf{0.79 \pm 0.09}$	0.72 ± 0.09
	**ENNUS**	$\mathbf{0.67 \pm 0.08}$	0.65 ± 0.1	0.62 ± 0.12	0.73 ± 0.13	$\mathbf{0.73 \pm 0.09}$
	**CNNUS**	0.65 ± 0.07	0.62 ± 0.09	0.59 ± 0.12	0.7 ± 0.13	0.69 ± 0.1
	**RandOS**	0.63 ± 0.07	0.68 ± 0.12	$\mathbf{0.63 \pm 0.1}$	$\mathbf{0.79 \pm 0.1}$	0.71 ± 0.09
	**SMOTE**	0.65 ± 0.08	0.66 ± 0.11	0.62 ± 0.11	0.76 ± 0.11	0.71 ± 0.1
	**BLSMOTE**	0.65 ± 0.07	0.65 ± 0.1	0.62 ± 0.1	0.75 ± 0.1	0.72 ± 0.08
	**ADASYN**	0.65 ± 0.07	0.64 ± 0.1	0.62 ± 0.1	0.75 ± 0.11	0.72 ± 0.09

	**SMOTETomek**	0.65 ± 0.07	0.66 ± 0.11	0.62 ± 0.11	0.76 ± 0.11	0.72 ± 0.09
	**SMOTEENN**	0.63 ± 0.08	0.67 ± 0.11	0.62 ± 0.1	**0.79 ± 0.11**	0.7 ± 0.11
	**Baseline**	0.65 ± 0.07	**0.73 ± 0.12**	**0.65 ± 0.1**	0.81 ± 0.09	0.76 ± 0.09
**Poly3PCA (n = 32)**	**RandUS**	**0.7 ± 0.08**	0.67 ± 0.11	**0.65 ± 0.13**	0.75 ± 0.14	0.76 ± 0.1
	**TomekUS**	0.65 ± 0.07	**0.73 ± 0.12**	**0.65 ± 0.1**	**0.82 ± 0.08**	0.76 ± 0.09
	**ENNUS**	0.69 ± 0.07	0.69 ± 0.11	**0.65 ± 0.11**	0.77 ± 0.11	**0.77 ± 0.08**
	**CNNUS**	0.69 ± 0.06	0.65 ± 0.09	0.63 ± 0.12	0.74 ± 0.13	0.74 ± 0.08
	**RandOS**	0.64 ± 0.07	0.72 ± 0.13	0.64 ± 0.1	0.81 ± 0.09	0.76 ± 0.1
	**SMOTE**	0.67 ± 0.08	0.69 ± 0.11	**0.65 ± 0.11**	0.79 ± 0.11	0.76 ± 0.1
	**BLSMOTE**	0.67 ± 0.08	0.69 ± 0.12	**0.65 ± 0.11**	0.79 ± 0.1	**0.77 ± 0.09**
	**ADASYN**	0.67 ± 0.08	0.68 ± 0.11	0.64 ± 0.11	0.78 ± 0.11	0.76 ± 0.09
	**SMOTETomek**	0.67 ± 0.07	0.69 ± 0.12	**0.65 ± 0.11**	0.79 ± 0.11	0.76 ± 0.1
	**SMOTEENN**	0.65 ± 0.07	0.72 ± 0.12	**0.65 ± 0.1**	0.81 ± 0.1	0.75 ± 0.1
	**Baseline**	0.63 ± 0.07	**0.69 ± 0.12**	**0.63 ± 0.09**	**0.81 ± 0.07**	0.73 ± 0.1
**rbfPCA (n = 8)**	**RandUS**	**0.67 ± 0.09**	0.61 ± 0.07	0.59 ± 0.11	0.69 ± 0.11	**0.74 ± 0.1**
	**TomekUS**	0.62 ± 0.07	0.68 ± 0.12	**0.63 ± 0.09**	0.8 ± 0.08	0.73 ± 0.1
	**ENNUS**	**0.67 ± 0.08**	0.63 ± 0.09	0.61 ± 0.11	0.72 ± 0.11	**0.74 ± 0.1**
	**CNNUS**	0.66 ± 0.08	0.62 ± 0.09	0.59 ± 0.12	0.68 ± 0.13	0.72 ± 0.11
	**RandOS**	0.63 ± 0.08	0.66 ± 0.1	0.62 ± 0.1	0.78 ± 0.09	0.72 ± 0.11
	**SMOTE**	0.65 ± 0.09	0.63 ± 0.09	0.61 ± 0.1	0.74 ± 0.1	0.72 ± 0.11
	**BLSMOTE**	0.64 ± 0.09	0.62 ± 0.08	0.6 ± 0.1	0.72 ± 0.1	0.71 ± 0.1
	**ADASYN**	0.65 ± 0.08	0.62 ± 0.08	0.6 ± 0.1	0.72 ± 0.1	0.72 ± 0.09
	**SMOTETomek**	0.64 ± 0.08	0.63 ± 0.09	0.61 ± 0.1	0.74 ± 0.1	0.72 ± 0.11
	**SMOTEENN**	0.63 ± 0.08	0.65 ± 0.1	0.62 ± 0.09	0.78 ± 0.09	0.71 ± 0.11
	**Baseline**	0.65 ± 0.06	**0.75 ± 0.12**	0.66 ± 0.08	**0.84 ± 0.07**	0.76 ± 0.09
**rbfPCA (n = 16)**	**RandUS**	**0.7 ± 0.08**	0.66 ± 0.1	0.65 ± 0.12	0.75 ± 0.12	0.76 ± 0.1
	**TomekUS**	0.64 ± 0.06	**0.75 ± 0.12**	0.66 ± 0.08	**0.84 ± 0.07**	0.76 ± 0.09
	**ENNUS**	**0.7 ± 0.07**	0.69 ± 0.12	**0.67 ± 0.11**	0.79 ± 0.09	**0.78 ± 0.08**
	**CNNUS**	0.69 ± 0.07	0.65 ± 0.1	0.63 ± 0.11	0.74 ± 0.11	0.74 ± 0.09
	**RandOS**	0.67 ± 0.06	0.73 ± 0.12	**0.67 ± 0.09**	0.83 ± 0.08	0.77 ± 0.09
	**SMOTE**	0.68 ± 0.07	0.69 ± 0.11	0.66 ± 0.1	0.8 ± 0.09	0.76 ± 0.1
	**BLSMOTE**	0.68 ± 0.07	0.67 ± 0.1	0.65 ± 0.1	0.78 ± 0.09	0.76 ± 0.09
	**ADASYN**	0.69 ± 0.07	0.67 ± 0.1	0.66 ± 0.1	0.78 ± 0.1	0.76 ± 0.09
	**SMOTETomek**	0.68 ± 0.07	0.69 ± 0.11	0.66 ± 0.1	0.8 ± 0.1	0.76 ± 0.09
	**SMOTEENN**	0.66 ± 0.07	0.73 ± 0.12	**0.67 ± 0.09**	0.82 ± 0.08	0.74 ± 0.1
	**Baseline**	0.64 ± 0.07	**0.77 ± 0.1**	0.66 ± 0.08	**0.85 ± 0.06**	**0.79 ± 0.09**
**rbfPCA (n = 32)**	**RandUS**	**0.71 ± 0.07**	0.67 ± 0.1	0.67 ± 0.1	0.78 ± 0.08	**0.79 ± 0.09**
	**TomekUS**	0.65 ± 0.06	**0.77 ± 0.1**	0.66 ± 0.08	**0.85 ± 0.06**	**0.79 ± 0.09**
	**ENNUS**	0.7 ± 0.06	0.7 ± 0.11	0.68 ± 0.08	0.82 ± 0.05	**0.79 ± 0.08**
	**CNNUS**	**0.71 ± 0.06**	0.67 ± 0.1	0.66 ± 0.11	0.77 ± 0.09	0.78 ± 0.07
	**RandOS**	0.68 ± 0.06	0.75 ± 0.11	0.68 ± 0.08	**0.85 ± 0.05**	**0.79 ± 0.08**
	**SMOTE**	0.69 ± 0.07	0.71 ± 0.1	0.68 ± 0.08	0.83 ± 0.06	**0.79 ± 0.08**
	**BLSMOTE**	0.69 ± 0.06	0.69 ± 0.1	0.67 ± 0.08	0.81 ± 0.06	**0.79 ± 0.08**

	**ADASYN**	0.7 ± 0.05	0.68 ± 0.09	0.67 ± 0.08	0.81 ± 0.07	$\mathbf{0.79 \pm 0.07}$
	**SMOTETomek**	0.69 ± 0.06	0.71 ± 0.1	0.68 ± 0.08	0.83 ± 0.05	$\mathbf{0.79 \pm 0.07}$
	**SMOTEENN**	0.67 ± 0.07	0.75 ± 0.11	$\mathbf{0.69 \pm 0.08}$	$\mathbf{0.85 \pm 0.06}$	0.78 ± 0.08
**sigPCA (n = 8)**	**Baseline**	0.62 ± 0.08	0.69 ± 0.11	0.62 ± 0.11	0.79 ± 0.12	0.73 ± 0.1
	**RandUS**	$\mathbf{0.68 \pm 0.09}$	0.66 ± 0.11	0.63 ± 0.14	0.74 ± 0.15	0.74 ± 0.11
	**TomekUS**	0.62 ± 0.08	0.69 ± 0.11	0.62 ± 0.11	0.79 ± 0.12	0.73 ± 0.1
	**ENNUS**	0.67 ± 0.07	0.67 ± 0.1	0.63 ± 0.12	0.76 ± 0.13	0.74 ± 0.09
	**CNNUS**	0.67 ± 0.08	0.63 ± 0.08	0.61 ± 0.13	0.72 ± 0.14	0.72 ± 0.11
	**RandOS**	0.63 ± 0.08	$\mathbf{0.71 \pm 0.12}$	0.63 ± 0.11	$\mathbf{0.8 \pm 0.12}$	0.74 ± 0.1
	**SMOTE**	0.65 ± 0.08	0.69 ± 0.12	$\mathbf{0.64 \pm 0.12}$	0.78 ± 0.12	0.74 ± 0.09
	**BLSMOTE**	0.65 ± 0.08	0.68 ± 0.11	0.63 ± 0.12	0.77 ± 0.12	0.74 ± 0.1
	**ADASYN**	0.66 ± 0.09	0.67 ± 0.11	$\mathbf{0.64 \pm 0.12}$	0.77 ± 0.13	$\mathbf{0.75 \pm 0.1}$
	**SMOTETomek**	0.65 ± 0.08	0.69 ± 0.12	$\mathbf{0.64 \pm 0.12}$	0.79 ± 0.12	0.74 ± 0.1
	**SMOTEENN**	0.62 ± 0.09	0.7 ± 0.12	0.63 ± 0.11	0.79 ± 0.12	0.73 ± 0.1
**sigPCA (n = 16)**	**Baseline**	0.65 ± 0.07	0.73 ± 0.12	0.65 ± 0.1	0.81 ± 0.1	0.77 ± 0.09
	**RandUS**	$\mathbf{0.7 \pm 0.09}$	0.69 ± 0.11	0.66 ± 0.14	0.76 ± 0.15	0.79 ± 0.09
	**TomekUS**	0.65 ± 0.07	0.72 ± 0.12	0.65 ± 0.1	0.81 ± 0.1	0.77 ± 0.08
	**ENNUS**	0.69 ± 0.07	0.69 ± 0.12	0.66 ± 0.11	0.79 ± 0.11	0.78 ± 0.07
	**CNNUS**	$\mathbf{0.7 \pm 0.08}$	0.66 ± 0.1	0.64 ± 0.14	0.73 ± 0.15	0.76 ± 0.1
	**RandOS**	0.66 ± 0.07	$\mathbf{0.77 \pm 0.12}$	$\mathbf{0.67 \pm 0.1}$	$\mathbf{0.84 \pm 0.08}$	0.79 ± 0.08
	**SMOTE**	0.68 ± 0.08	0.73 ± 0.12	$\mathbf{0.67 \pm 0.11}$	0.82 ± 0.09	0.79 ± 0.08
	**BLSMOTE**	0.68 ± 0.08	0.73 ± 0.12	$\mathbf{0.67 \pm 0.11}$	0.82 ± 0.09	$\mathbf{0.8 \pm 0.09}$
	**ADASYN**	0.68 ± 0.09	0.73 ± 0.12	$\mathbf{0.67 \pm 0.12}$	0.82 ± 0.09	$\mathbf{0.8 \pm 0.08}$
	**SMOTETomek**	0.67 ± 0.08	0.73 ± 0.13	$\mathbf{0.67 \pm 0.11}$	0.82 ± 0.1	0.79 ± 0.08
	**SMOTEENN**	0.66 ± 0.07	0.75 ± 0.13	0.66 ± 0.1	0.83 ± 0.08	0.78 ± 0.08
**sigPCA (n = 32)**	**Baseline**	0.64 ± 0.08	0.72 ± 0.12	0.63 ± 0.11	0.8 ± 0.12	0.77 ± 0.1
	**RandUS**	$\mathbf{0.73 \pm 0.08}$	0.69 ± 0.11	$\mathbf{0.67 \pm 0.14}$	0.76 ± 0.15	0.8 ± 0.1
	**TomekUS**	0.64 ± 0.08	0.72 ± 0.12	0.64 ± 0.11	0.8 ± 0.12	0.77 ± 0.1
	**ENNUS**	0.69 ± 0.07	0.7 ± 0.11	0.66 ± 0.12	0.78 ± 0.12	0.78 ± 0.09
	**CNNUS**	0.72 ± 0.08	0.65 ± 0.08	0.62 ± 0.14	0.7 ± 0.16	0.78 ± 0.11
	**RandOS**	0.61 ± 0.07	$\mathbf{0.76 \pm 0.14}$	0.62 ± 0.1	$\mathbf{0.82 \pm 0.1}$	0.8 ± 0.1
	**SMOTE**	0.66 ± 0.07	0.74 ± 0.14	0.66 ± 0.12	$\mathbf{0.82 \pm 0.11}$	0.8 ± 0.09
	**BLSMOTE**	0.66 ± 0.08	0.74 ± 0.14	0.66 ± 0.12	$\mathbf{0.82 \pm 0.1}$	$\mathbf{0.81 \pm 0.09}$
	**ADASYN**	0.66 ± 0.09	0.73 ± 0.14	0.66 ± 0.13	0.81 ± 0.12	0.8 ± 0.1
	**SMOTETomek**	0.66 ± 0.08	0.74 ± 0.14	0.66 ± 0.12	0.81 ± 0.12	0.8 ± 0.1
	**SMOTEENN**	0.63 ± 0.08	0.75 ± 0.14	0.64 ± 0.11	0.81 ± 0.11	0.79 ± 0.1
**cosPCA (n = 8)**	**Baseline**	0.63 ± 0.08	$\mathbf{0.71 \pm 0.13}$	0.63 ± 0.11	$\mathbf{0.8 \pm 0.1}$	0.75 ± 0.12
	**RandUS**	$\mathbf{0.69 \pm 0.09}$	0.67 ± 0.12	$\mathbf{0.64 \pm 0.14}$	0.74 ± 0.14	$\mathbf{0.77 \pm 0.12}$
	**TomekUS**	0.63 ± 0.08	$\mathbf{0.71 \pm 0.13}$	$\mathbf{0.64 \pm 0.11}$	$\mathbf{0.8 \pm 0.1}$	0.75 ± 0.12
	**ENNUS**	0.68 ± 0.08	0.68 ± 0.13	$\mathbf{0.64 \pm 0.12}$	0.77 ± 0.11	$\mathbf{0.77 \pm 0.1}$
	**CNNUS**	0.67 ± 0.09	0.64 ± 0.11	0.61 ± 0.14	0.7 ± 0.14	0.72 ± 0.12
	**RandOS**	0.64 ± 0.08	$\mathbf{0.71 \pm 0.13}$	$\mathbf{0.64 \pm 0.11}$	0.79 ± 0.11	0.76 ± 0.12
	**SMOTE**	0.66 ± 0.08	0.69 ± 0.13	$\mathbf{0.64 \pm 0.12}$	0.77 ± 0.12	0.76 ± 0.12

	**BLSMOTE**	0.67 ± 0.07	0.68 ± 0.12	$\mathbf{0.64 \pm 0.11}$	0.77 ± 0.11	$\mathbf{0.77 \pm 0.11}$
	**ADASYN**	0.67 ± 0.08	0.68 ± 0.13	$\mathbf{0.64 \pm 0.12}$	0.76 ± 0.12	0.76 ± 0.11
	**SMOTETomek**	0.66 ± 0.08	0.69 ± 0.13	$\mathbf{0.64 \pm 0.12}$	0.77 ± 0.13	0.76 ± 0.12
	**SMOTEENN**	0.65 ± 0.08	0.7 ± 0.13	$\mathbf{0.64 \pm 0.11}$	0.79 ± 0.12	0.75 ± 0.12
**cosPCA (n = 16)**	**Baseline**	0.66 ± 0.07	$\mathbf{0.76 \pm 0.13}$	0.68 ± 0.1	0.84 ± 0.07	0.78 ± 0.11
	**RandUS**	$\mathbf{0.71 \pm 0.09}$	0.69 ± 0.12	0.67 ± 0.13	0.78 ± 0.12	$\mathbf{0.8 \pm 0.1}$
	**TomekUS**	0.66 ± 0.07	0.75 ± 0.13	0.67 ± 0.1	0.84 ± 0.07	0.78 ± 0.11
	**ENNUS**	0.7 ± 0.08	0.72 ± 0.12	$\mathbf{0.69 \pm 0.11}$	0.82 ± 0.08	$\mathbf{0.8 \pm 0.1}$
	**CNNUS**	0.7 ± 0.08	0.67 ± 0.11	0.64 ± 0.13	0.75 ± 0.12	0.77 ± 0.1
	**RandOS**	0.67 ± 0.07	$\mathbf{0.76 \pm 0.11}$	$\mathbf{0.69 \pm 0.09}$	$\mathbf{0.85 \pm 0.07}$	$\mathbf{0.8 \pm 0.1}$
	**SMOTE**	0.7 ± 0.08	0.73 ± 0.12	$\mathbf{0.69 \pm 0.1}$	0.83 ± 0.08	$\mathbf{0.8 \pm 0.1}$
	**BLSMOTE**	0.69 ± 0.07	0.72 ± 0.11	$\mathbf{0.69 \pm 0.08}$	0.84 ± 0.06	$\mathbf{0.8 \pm 0.08}$
	**ADASYN**	0.7 ± 0.07	0.71 ± 0.11	$\mathbf{0.69 \pm 0.09}$	0.83 ± 0.06	$\mathbf{0.8 \pm 0.09}$
	**SMOTETomek**	0.69 ± 0.08	0.73 ± 0.12	$\mathbf{0.69 \pm 0.1}$	0.83 ± 0.08	$\mathbf{0.8 \pm 0.1}$
	**SMOTEENN**	0.68 ± 0.07	0.75 ± 0.12	$\mathbf{0.69 \pm 0.09}$	0.84 ± 0.07	0.79 ± 0.1
**cosPCA (n = 32)**	**Baseline**	0.63 ± 0.07	0.78 ± 0.13	0.66 ± 0.09	$\mathbf{0.85 \pm 0.07}$	0.81 ± 0.1
	**RandUS**	$\mathbf{0.73 \pm 0.08}$	0.7 ± 0.12	$\mathbf{0.68 \pm 0.12}$	0.79 ± 0.11	$\mathbf{0.83 \pm 0.1}$
	**TomekUS**	0.63 ± 0.08	0.78 ± 0.13	0.66 ± 0.1	$\mathbf{0.85 \pm 0.06}$	0.81 ± 0.11
	**ENNUS**	0.68 ± 0.07	0.74 ± 0.12	$\mathbf{0.68 \pm 0.1}$	0.83 ± 0.07	0.82 ± 0.1
	**CNNUS**	$\mathbf{0.73 \pm 0.07}$	0.66 ± 0.1	0.64 ± 0.14	0.72 ± 0.14	0.81 ± 0.09
	**RandOS**	0.64 ± 0.07	$\mathbf{0.79 \pm 0.13}$	0.66 ± 0.09	$\mathbf{0.85 \pm 0.06}$	$\mathbf{0.83 \pm 0.1}$
	**SMOTE**	0.67 ± 0.08	0.75 ± 0.13	$\mathbf{0.68 \pm 0.1}$	0.84 ± 0.07	$\mathbf{0.83 \pm 0.1}$
	**BLSMOTE**	0.65 ± 0.07	0.74 ± 0.13	0.66 ± 0.09	0.84 ± 0.06	0.81 ± 0.09
	**ADASYN**	0.66 ± 0.07	0.74 ± 0.13	0.67 ± 0.1	0.83 ± 0.07	0.81 ± 0.1
	**SMOTETomek**	0.67 ± 0.08	0.75 ± 0.13	$\mathbf{0.68 \pm 0.1}$	0.84 ± 0.07	0.82 ± 0.1
	**SMOTEENN**	0.65 ± 0.08	0.77 ± 0.13	0.67 ± 0.1	$\mathbf{0.85 \pm 0.07}$	0.82 ± 0.1
